# Binding of Cholesterol to the N-Terminal Domain of the NPC1L1 Transporter: Analysis of the Epimerization-Related Binding Selectivity and Loop Mutations

**DOI:** 10.1021/acs.jcim.3c01319

**Published:** 2023-12-28

**Authors:** Aitor Valdivia, F. Javier Luque, Salomé Llabrés

**Affiliations:** † Departament de Nutrició, Ciències de l′Alimentació i Gastronomia, Facultat de Farmàcia i Ciències de l′Alimentació—Campus Torribera, Universitat de Barcelona, Prat de la Riba 171, 08921 Santa Coloma de Gramenet, Spain; ‡ Institut de Biomedicina (IBUB), Universitat de Barcelona, 08028 Barcelona, Spain; § Institut de Química Teòrica i Computacional (IQTCUB), Universitat de Barcelona, 08921 Barcelona, Spain

## Abstract

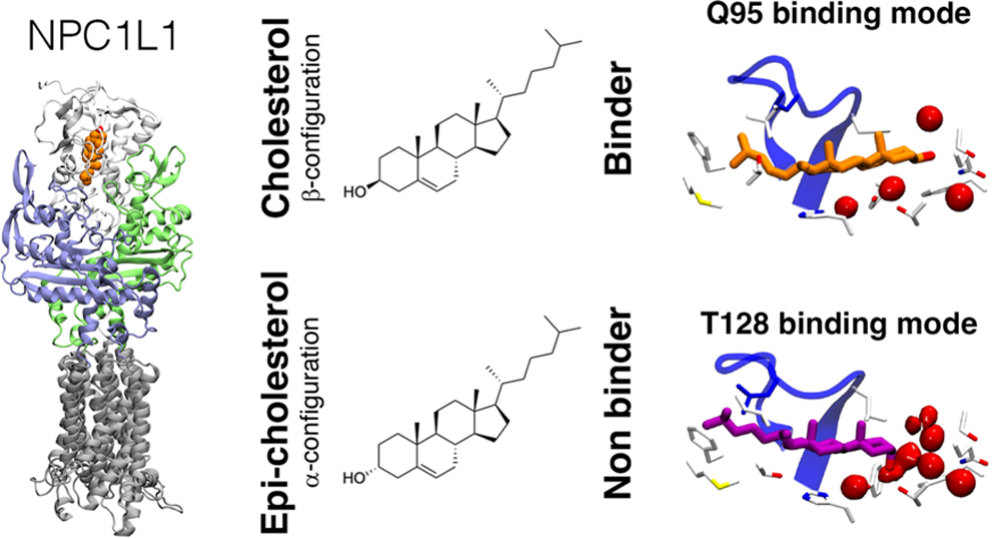

Cholesterol is a fat-like substance with a pivotal physiological relevance in humans, and its homeostasis is tightly regulated by various cellular processes, including the import in the small intestine and the reabsorption in the biliary ducts by the Niemann–Pick C1 Like 1 (NPC1L1) importer. NPC1L1 can mediate the absorption of a variety of sterols but strikingly exhibits a large sensitivity to cholesterol epimerization. This study examines the molecular basis of the epimerization-related selective binding of cholesterol by combining extended unbiased molecular dynamics simulations of the apo and holo species of the N-terminal domain of wild-type NPC1L1, in conjunction with relative binding free energy, umbrella sampling, and well-tempered metadynamics calculations. The analysis of the results discloses the existence of two distinct binding modes for cholesterol and epi-cholesterol. The former binds deeper in the cavity, forming key hydrogen-bond interactions with Q95, S56, and a water molecule. In contrast, epi-cholesterol is shifted ca. 3 Å to the mouth of the cavity and the transition to the Q95 site is prevented by an energetic barrier of 4.1 kcal·mol^–1^. Thus, the configuration of the hydroxyl group of cholesterol, together with the presence of a structural water molecule, is a key feature for effective absorption. Finally, whereas these findings may seemingly be challenged by single-point mutations that impair cholesterol transport but have a mild impact on the binding of cholesterol to the Q95 binding site, our results reveal that they have a drastic influence on the conformational landscape of the α8/β7 loop in the apo species compared to the wild-type protein. Overall, the results give support to the functional role played by the α8/β7 loop in regulating the access of ligands to NPC1L1, and hence to interpreting the impact of these mutations on diseases related to disruption of sterol absorption, paving the way to understanding certain physiological dysfunctions.

## Introduction

Cholesterol is an essential component of mammalian cell membranes and a key precursor of several metabolites, such as steroid hormones, vitamin D, and bile salts. It plays an important role in the rigidity and fluidity of the cell membrane and also in regulating the functional conformations of membrane proteins. The multiple physiological roles modulated by cholesterol demand a tight regulation of cholesterol homeostasis, which involves three main processes: endogenous biosynthesis, dietary absorption in the intestine, and net biliary excretion and reabsorption.^1,2^ Alterations in the metabolic pathways that control cholesterol homeostasis are implicated in the etiology of various diseases and particularly have been associated with a higher risk of cardiovascular disease, neurodegeneration, and cancer.^1,3,4^

**Figure 1:**
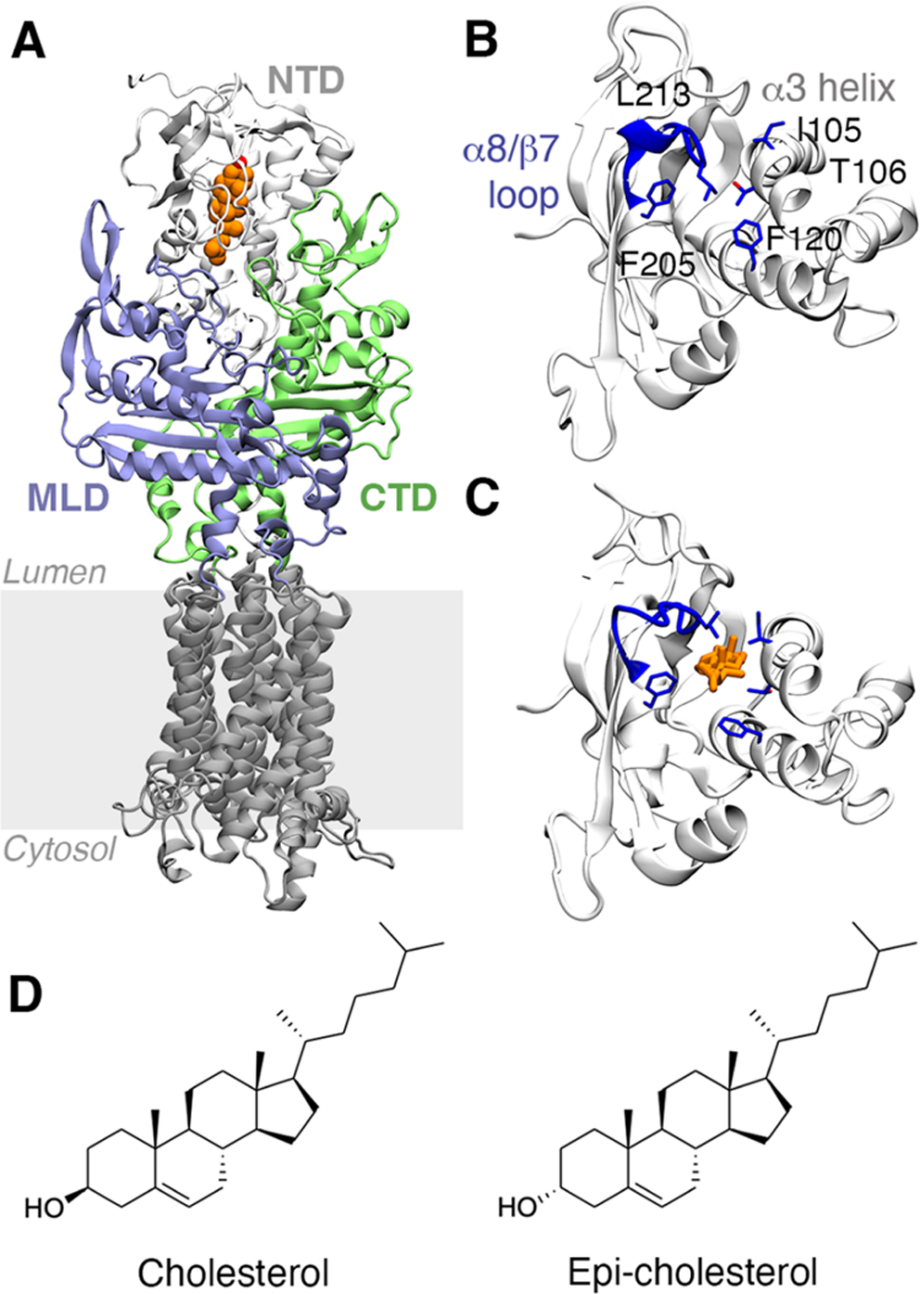
(A) Representation of the N-terminal (NTD), middle (MLD), and C-terminal (CTD) domains (shown as white, blue, and green cartoons, respectively) of NPC1L1 present in the luminal space and the transmembrane region. Detail of the entrance to the binding site of the NPC1L1-NTD in (B) the apo (X-ray structure; PDB 3QNT)^15^ and (C) cholesterol-bound state. These structures illustrate the open-closed conformational change of the α8/β7 loop, which regulates the access of sterols to the binding site in conjunction with residues I105, T106, F120, L213, and F205 (shown as blue sticks). The crystallographic position of cholesterol in NPC1 is shown as sticks (C atoms in orange). (D) Structure of cholesterol and epi-cholesterol.

The Niemann–Pick C1 Like 1 (NPC1L1) protein is the primary importer of cholesterol in the small intestine and the reabsorption of sterols in the biliary duct.^5^ NPC1L1 is also the molecular target of ezetimibe, which is one of the most prescribed drugs to lower high blood cholesterol levels.^6^ From a structural point of view, NPC1L1 consists of three large extracellular (N-terminal, middle-luminal, and C-terminal) domains and a large transmembrane region that comprises 13 helices (Figure 1A).^7^ The N-terminal domain (NTD) is free to unbind from the NPC1L1 core to capture cholesterol in extracellular media. Upon binding, the NTD folds over the other luminal domains, creating a continuous hydrophobic tunnel between the NTD and the membrane, thus enabling cholesterol to reach the cellular membrane.^7,8^ Mutations in the NPC1L1 gene are associated with lower cholesterol absorption, which results in lower coronary heart disease and infarction risk.^9^ Therefore, NPC1L1 inhibitors, such as ezetimibe,^10^ open avenues for the effective regulation of cholesterol levels in plasma in patients with hypercholesterolemia^11^ and sitosterolaemia^12^ and can be used as coadjuvants in combination chemotherapies.^13,14^

NPC1L1 imports selectively cholesterol as well as sterol precursors and vitamins.^5^ The molecular basis of its ligand specificity is encoded into the structural features of the NTD binding site.^16,17^ The X-ray structure of the apo NTD has been solved in its closed conformation, which prevents access to the binding site via the steric blockage by the side chain of L213 of the α8/β7 loop (Figure 1B). Although there is no X-ray structure for the cholesterol-bound NTD, details of the binding mode have been gained from the holo species of the homologous Niemann–Pick C1 (NPC1) protein (40% identity and 57% similarity; 41% identity and 64% similarity considering only the residues involved in the cholesterol-binding site), which has been solved in complex with cholesterol and 25-hydroxycholesterol.^17^ Whereas the NPC1L1 structure shows an empty binding site, the structures of cholesterol-bound NPC1 reveal the identification of the binding mode of this compound as well as the hydration pattern within the binding site (Figures S1 and S2). Inspection of these NPC1 structures shows that residue Q200, equivalent to L213 in NPC1L1, is exposed to the solvent and interacts with helix α3, stabilizing the open conformation that enables access to the binding pocket (Figure 1C). Therefore, it has been hypothesized that the loop α8/β7 plays a key role in the gating mechanism that regulates the sterol binding to NPC1L1.^16,17^

The structural similarity between the homologous domains of NPC1 and NPC1L1 paves the way to identifying the molecular basis of the selective binding of cholesterol and related sterols to the NPC1L1 transporter. In this regard, the drastic reduction in transport efficiency triggered upon epimerization of the hydroxyl group bound at carbon 3 in cholesterol (Figure 1D) is in contrast with the effective competition observed for other natural sterols that retain the β configuration of the hydroxyl group in cholesterol.^15,18^ To gain insight into the molecular basis of the epimerization-related selective binding of cholesterol to NPC1L1, this study reports a series of extensive molecular dynamics (MD) simulations that have been used to explore the binding mode of cholesterol and epi-cholesterol. The analysis of the trajectories reveals the existence of distinct binding modes to the NTD domain of the NPC1L1. Free energy calculations have been used to examine the structural stability of the binding mode of these compounds and to estimate the differences in binding affinity between cholesterol and epi-cholesterol. The binding mode of cholesterol has been confirmed for different sterols that retain the β configuration of the hydroxyl group. Finally, the effect of distinct mutations on the binding of cholesterol is discussed by considering the impact of the mutated variants on the structural features implicated in ligand binding. The results highlight the subtle interplay between different factors in modulating the binding mode and affinity of sterols for NPC1L1, including the epimerization at the C3 position, the role of water molecules in assisting the stability of the binding mode, and the impact of mutations on the accessibility to the binding pocket.

## Methods

### Setup of the Systems

The NPC1L1-NTD domain was modeled from the X-ray structure solved by Kwon et al. (PDB entry: 3QNT, resolution 2.83 Å),^15^ which corresponds to the closed conformation that occludes the sterol binding pocket from solvent. The crystallographic structure of cholesterol in the NTD of the homologous protein human NPC1 (PDB entry: 3GKI, resolution 1.80 Å)^18^ was used as a template for the binding mode of sterols in the ligand-bound complexes of NPC1L1-NTD. Given the almost perfect overlay of the residues that shape the binding pocket in the NPC1–cholesterol complex and those present in the NPC1L1-NTD, cholesterol was placed within the binding site of NPC1L1 by translating the coordinates of the ligand bound to NPC1. Indeed, only a slight steric overlap was detected between the side chain of L213 and the aliphatic chain of cholesterol. Nevertheless, it was alleviated through energy minimization of the system, which was the first step of the heating and equilibration protocol. The coordinates of the refined cholesterol–NPC1L1 system were subsequently used to model the binding of the sterol compounds.

The systems were solvated using a dodecahedral box of TIP3P water molecules accounting for a 12 Å spacing between the protein and the box edge. Counterions (Na^+^ and Cl^–^) were added to maintain the neutrality of the simulated system at an ionic concentration of 0.15 M following the SPLIT method.^19^ The simulation systems account for a total of ∼53,500 atoms in a simulation box of 88 × 83 × 72 Å^3^. No crystallographic waters were included in the initial setup, as none are reported in any of the experimentally solved structures of the NPC1L1-NTD (Tables S1 and S2). Nevertheless, a 15 ns long equilibration protocol was used to ensure proper hydration of the binding site (see the next section for additional details).

The amberff14sb force field^20^ was used for the protein, and the sterol molecules were parametrized using the Lipid14 force field.^21^ Joung and Cheatham III parameters were used for the counterions,^22^ and the TIP3P model^23^ was used for the solvent. Partial atomic charges for the ligands were derived using the RESP protocol^24^ at the HF/6-31G(d) level of theory, as calculated with Gaussian09.^25^ Finally, the parameters were converted to Gromacs file-format using the AnteChamber PYthon Parser interfacE (ACPYPE) tool.^26^

### Molecular Dynamics Simulations

Each system was minimized using 15,000 steps of steepest descent, followed by 5000 steps of conjugate gradient algorithm. Then, each system was gradually equilibrated in 9 steps. The systems were heated in the NVT ensemble from 100 to 300 K in three stages of 1 ns (100–150, 150–250, 250–300 K) with 5 kcal·mol^–1^ positional restraints on the backbone atoms of the protein and all of the atoms of the ligand. Subsequently, the density of the system was equilibrated for 12 ns in the NPT ensemble (pressure: 1 bar, T: 300 K). During the last 5 ns of the equilibration run, the positional restraints were slowly released, decreasing 1.0 kcal·mol^–1^ per ns.

Production runs encompassed 4 replicas of 0.5 μs per system, accounting for a total simulation time of 2 μs per system (for a total of 28 μs of simulation time). Temperature control was achieved using a V-rescale thermostat.^27^ Pressure control was maintained by using the Parrinello–Rahman method. All bonds involving hydrogen atoms were constrained by the LINCS algorithm^28^ to allow for a time step of 2 fs. All simulations were performed with the Gromacs 2022.4 package.^29^

### Molecular Mechanics Poisson–Boltzmann Surface Area (MM-PBSA) Calculations

The binding free energy of sterol molecules to NPC1L1-NTD was estimated using MM-PBSA calculations with the MMPBSA.py script available in AmberTools22.^30^ The theoretical details of the method are described in detail elsewhere.^31^ For each sterol-bound complex, a total of 400 configurations (100 taken from each independent replicate) were extracted and preprocessed to include key structural water present in most of the simulations (see below) as part of the receptor. Dielectric constants of 2 and 80 were used for the interior of the protein and the bulk solvent, respectively, in conjunction with an ionic strength of 0.15 M.^32^ No entropic contributions were calculated since the final aim was to estimate the relative binding free energies (RBFE) of structurally related, largely rigid ligands that adopt a similar arrangement in the binding pocket.

The *cpptraj* package was used for the analysis of the simulated systems. The volume of the binding pocket was calculated with MDPocket.^33^

### Binding Free Energy Calculations

The relative binding free energy (RBFE)^34^ between sterols was determined through alchemical transformations where a ligand (L1) is converted into a structurally related analogue (L2) in both the protein-bound complex and in the unbound state in aqueous solution (eq 1). Accordingly, independent sets of simulations were carried out for the transformation between L1 and L2 in water and in the protein.
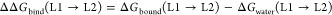
1

The transformation from L1 to L2 was split in a set of windows, where λ = 0 and λ = 1 correspond to L1 and L2, respectively, and the intermediate λ values correspond to a linear interpolation between the parameters of the initial and final systems.^35^ A dual topology alchemical perturbation protocol was employed by using 21 evenly spaced λ-windows. To smoothly transform L1 to L2, we applied soft-core potentials using soft-core electrostatics^36^ as described in AMBER22^35^ to the ring A of the sterol core. All of the λ simulations were run for 6 ns, accounting for a total of 126 ns per transformation (12 transformations, over 1.5 μs of RBFE simulations). The Thermodynamic Integration (TI) estimator, as implemented in *alchemlyb*,^37^ was used to estimate the free energy change for each λ simulation. All simulations were carried out with the GPU-accelerated TI implementation^34,38^ of Amber22.

The initial coordinates for each binding mode were extracted from the unbiased MD runs. All of the systems were carefully equilibrated with the dual topology using the TI code in each transformation with λ = 0.5. Each system was first heated in the NVT ensemble from 5 to 300 K (200 ps) and subsequently equilibrated at 1 bar in the NPT ensemble (300 ps). During the equilibration protocol, the heavy atoms of the protein and ligand were restrained with positional restraints (5 kcal·mol^–1^). Each λ state was then simulated in the NVT ensemble at 300 K by using the Langevin thermostat and the SHAKE algorithm for bonds involving hydrogen atoms.

Each simulation was repeated three times to assess reproducibility of the free energy changes. The resulting binding free energies were averaged, and their standard deviations were used for the error analysis. Additionally, we calculated the cycle closure error to assess the convergence of the simulations. Convergence was reached when the cycle closure errors were below 0.3 kcal·mol^–1^.

### Umbrella Sampling (US) Calculations

To estimate the potential of mean force (PMF) for the transition of epi-cholesterol between the two binding modes in the ligand cavity, a series of five steered MD simulations were performed to sample the shift from the T128 anchoring site to the Q95 binding one.^39^ To this end, the distance between the N atom of the Q95 side chain amide group and the O atom of epi-cholesterol was chosen as a transition coordinate. Steered MD simulations were started by using snapshots taken from the unbiased MD runs, where the T128 binding mode was stable. The transition of epi-cholesterol between the two anchoring sites was driven by using a speed of 0.5 Å·ns^–1^ in conjunction with a force constant of 500 kcal·mol^–1^ Å^–2^ (hence using the stiff-spring approximation).

The trajectory that showed lower work profiles was selected for the umbrella sampling (US) calculations, which were performed to estimate the potential of mean force (PMF) for the transition between the Q95 and T128 binding modes of epi-cholesterol. The distance between the N atom of the Q95 side chain and the O atom of the epi-cholesterol molecule was varied within a range of 2.5–8 Å and an interval of 0.5 Å, accounting for a total of 12 bins. The initial coordinates of each bin were selected by choosing the snapshot of the steered MD trajectories with the closest value to the ideal transition coordinate. Harmonic potential restraints of 1 kcal·mol^–1^ Å^–2^ were applied to avoid deviations from the target values along the transition coordinate that connects the two binding states. Each bin was run for 6 ns using the same conditions described in the MD section. Values of the reaction coordinates were stored every 10 fs for postprocessing. The WHAM method^40^ was used to obtain the PMF profile as implemented in the GROMACS package (*gmx wham*), and the error bars were computed by bootstrapping 100 times.

### Well-Tempered Metadynamics Simulations

To further check the results obtained from the US calculations, multiple-walkers well-tempered metadynamics simulations (MW-WTM)^41−43^ were used to examine the transition of epi-cholesterol between the two binding modes defined by interactions with Q95 and T128 in the ligand cavity. Two collective variables were defined to guide conformational sampling. The first collective variable (CV1) accounts for the difference between the distances of the H-bonds formed by the oxygen atom of epi-cholesterol with the side chains of Q95 and T128 (the Q95 and T128 binding modes are mapped onto the negative and positive values of CV1, respectively). The second collective variable (CV2) was chosen to follow the conformational fluctuations of the S-entrance by measuring the distance between the center-of-mass (COM) of residues 212–214 of the α8β7 loop and the COM of residues 104–106 of α-helix 3. Lower and upper walls were placed to keep the distances between the O atom of epi-cholesterol and the N/O atom of the Q95/T128 side chain within the range of 2–10 Å. Likewise, CV2 was maintained between 7 and 17 Å to sample the conformational space of the S-entrance in the unbiased MD simulations and to avoid drastic changes (i.e., unfolding) in the α8β7 loop. Six independent walkers^44^ were used to contribute simultaneously to the same history-dependent bias potential and to explore the free energy surface (FES). The deposition rate of the Gaussian biasing terms was set to 1 ps, their initial height to 0.4 kcal mol^–1^, and a bias factor of 15. Initial positions for each walker were set along the path that connects the Q95 and T128 binding modes obtained from US calculations. The system was simulated for a total of 1.2 μs, and the bias potential was updated every 0.2 ps. The minimum energy path between the Q95 and T129 binding modes was obtained using the string method^45,46^ over the FES.

### Principal Component Analysis (PCA)

To explore the conformational landscape of the S-opening in the NPC1L1-NTD upon cholesterol binding and absorption impairing mutations, we performed principal component analysis (PCA) onto the apo and cholesterol-bound trajectories of wild-type and mutant domains. We only included the main-chain atoms (C, Cα, N, and O atoms) of the α8/β7 loop (from Q206 to L216) and the α3 helix (from A93 to L110). The trajectories were properly aligned to the equilibrated structure of NPC1L1-NTD in the apo state. Then, the eigenvalues and eigenvectors were calculated by diagonalizing the mass-weighted covariance matrix of the system using the *gmx covar* and *gmx anaeig* modules of the GROMACS 2022.4 package.

The relative population of each conformational state sampled for the apo- and cholesterol-bound systems was estimated after projection in the space defined by the first two principal components (PCs), which account for 66% of the total structural variance (see below). To this end, we used the k-means clustering with 100 clusters to discretize the PC space, and then these clusters were further classified into eight conformational states (five for the apo and three for the cholesterol-bound systems) using the Robust Perron Cluster Analysis method (PCCA+)^47^ as implemented in the PyEMMA 2.0 module.^48^ The error bars of these populations were obtained by bootstrapping 5 times. Fifty structures of each of these conformational states were extracted for visualization and structural analysis purposes.

## Results and Discussion

### Conformational Changes of the α8/β7 Loop of NPC1L1 upon Ligand

The NTD domains of both NPC1L1 and NPC1 have a common protein fold, as noted in a positional root-mean-square deviation (RMSD) of 1.6 Å determined for the Cα atoms (Figures S1 and S2); however, comparison of their structures highlights the subtle but relevant rearrangement of the α8/β7 loop, which affects the accessibility to the binding pocket. In the NPC1L1-NTD, the side chain of L213 is buried into the entrance of the binding site, thus preventing ligand binding. In contrast, residue Q200 of the α8/β7 loop of NPC1-NTD, equivalent to L213 of NPC1L1-NTD, interacts with N86 of the α3 helix and stabilizes the open conformation.^17^ This structural rearrangement enlarges the entrance to the binding pocket in NPC1 compared to NPC1L1, as noted in the width of the cavity mouth, which increases from 7.6 Å in NPC1L1-NTD (X-ray structure 3QNT) to 9.3 Å in NPC1-NTD (X-ray structure 3GKI) measured as the distance between the C_β_ atoms of L213 and T106 of NPC1L1-NTD, and the C_β_ atoms of Q200 and P90 of NPC1-NTD.

The structural stability of the apo- and cholesterol-bound states of NPC1L1-NTD was examined by means of extensive unbiased MD simulations. Four independent replicates, each covering 0.5 μs, were run for each system, thus leading to a cumulative simulation time of 2 μs for the apo and cholesterol-bound forms of NPC1L1-NTD. Stable RMSD profiles were obtained in the four replicates run for both apo and holo systems (Figures S3 and S4)

**Figure 2:**
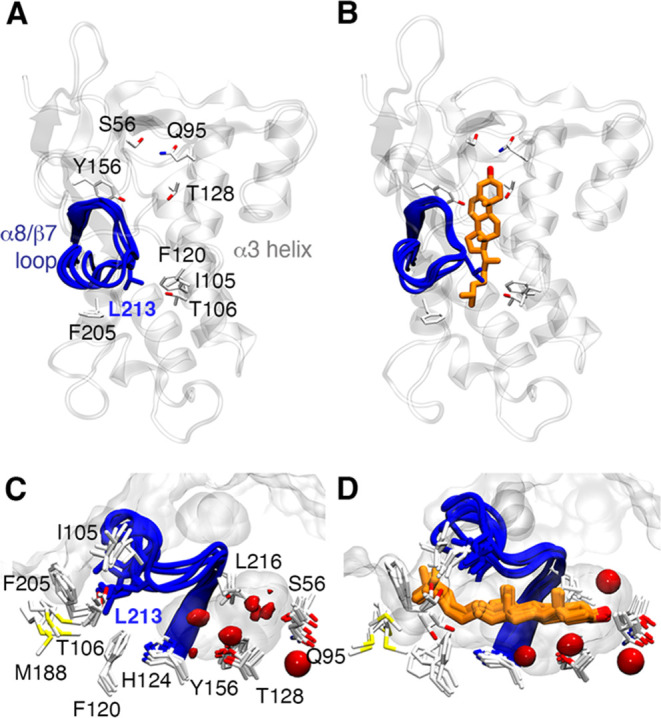
Superposition of the final structures of the MD trajectories of the apo (A, C) and cholesterol-bound states (B, D). (A, B) The conformations sampled by the α8/β7 loop are shown in a blue cartoon. (C, D) Shape and solvation pattern of the binding site of NPC1L1-NTD for the apo and cholesterol-bound states. The protein surface is shown as a gray surface. The red isosurface accounts for 50% of water occupancy in the binding site during the last 100 ns of each replicate (aggregated 400 ns of simulation time).

Our simulations confirm that, in the apo state, the side chain of L213 interacts with L103, T106, F120, and F205, thus forming a stable hydrophobic cluster that keeps the closed conformation, which hinders the entrance to the binding site (Figure 2; see also Figure S5), also known as S-opening. These residues bury the side chain of L213 in the binding site close to the phenyl ring of F120 for most of the simulation time (average distance of 5.4 ± 0.6 Å; present in 58% of the simulation time). This conformation is further stabilized by a H-bond interaction between the backbone of L213 and S102 on the α3 helix (average distance of 2.7 ± 0.1 Å; 45% of the simulation time), which brings the α8/β7 loop upon the α3 helix and tightens this hydrophobic cluster. This causes a slight distortion from the X-ray structure, as the RMSD of the Cα atoms of the α8/β7 loop is on average 2.0 ± 0.9 Å, accompanied by a decrease in the width of the cavity mouth, which amounts on average to 6.5 ± 0.6 Å (average distance from the Cβ atoms of the L213 and T106), compared to the X-ray structure (distance of 7.6 Å). The side chain of L213 can also adopt a distinct conformation that enables the interaction with I105 (average distance of 5.5 ± 0.3 Å; 37% of the simulation time) but without altering the entrance of the binding site (6.3 ± 0.8 Å). Altogether, the two conformations adopted by L213 restrict the volume of the binding site, which amounts to 374 ± 4 Å^3^.

Despite the hydrophobic nature of the cavity, between 6 and 16 water molecules were found in the binding site, with an average number of 11 ± 2 waters for the four replicates (Figure S6). The access of water molecules to the binding site occurs through the W opening, which is shaped by the α1 and α2 helices that lie on top of the cavity. Also, the binding pocket has a subset of polar residues composed of Q95, S56, Y156, T128, and H124 (Figure 2) that interact with a network of water molecules that fill the binding pocket. This network overlaps nicely with the waters detected in the crystallographic structure of the apo state of the NPC1 (Figure S7).^17^

The binding of cholesterol requires the conformational rearrangement of the side chain of L213, which interacts with the ligand’s side chain (average distance of 4.9 ± 0.1 and 5.2 ± 0.1 Å with the carbon atom of the methyl group in position C18 of the sterol moiety and the first carbon atom of the aliphatic side chain, respectively). This interaction is facilitated by the fact that L213 adopts a conformation that mimics the arrangement of Q200 in NPC1-NTD, where its side chain points to I105 in the α3 helix (Figure S7). The presence of cholesterol in the binding site forces the open conformation of the pocket, which is stable and exhibits small fluctuations in the four replicates (Figure 2; see Figure S3) in agreement with previous studies.^16^ Thus, the average RMSD determined for the Cα atoms of the α8/β7 loop relative to the X-ray structure of NPC1-NTD is 2.3 ± 0.1 Å. Compared to the apo species, the expansion of the cavity is reflected in the width of the mouth of the binding site, which on average amounts to 8.3 ± 0.5 Å, and in the enlargement of the cavity volume (average value of 1031 ± 18 Å^3^), it being around 3-fold larger than the volume determined for the apo species.

The crystallographic binding mode of cholesterol was fully maintained in the simulations of the complex with NPC1L1-NTD (Figure 2). The hydroxyl moiety of cholesterol can establish three H-bond interactions with neighboring residues: the amide NH of Q95, the hydroxyl group of S56, and a water-mediated interaction with the backbone of Q54 [let us note that this water molecule is also present in the experimental structure of NPC1 (Figure S7)].^17^ While the H-bond interactions with Q95 are stable along all of the replicates (average distance of 2.9 ± 0.1 Å; 66% of the simulation time), the S56 side chain fluctuates between its interaction with the ligand (average distance of 2.8 ± 0.1 Å; detected in 17% of the simulation time) and the backbone carbonyl oxygen of D37. The hydrophobic tetracyclic system of cholesterol interacts with residues L99, L103, and H124 of the α-helices of the NTD core and residues A214 and P215 of the α8/β7 loop. Finally, the aliphatic chain is partially exposed to the bulk solvent at the S-opening, where it interacts with L213 and F205 (Figure S8). Hereafter, given the increased stability of the interaction with Q95, this binding mode will be referred to as the Q95 binding mode.

The binding of cholesterol displaces most of the water molecules present in the binding site, which, however, still contains an average number of 5 ± 1 water molecules (Figures S7 and S9). The 50% occupancy isocontour of the water density accounts for the water wire that extends from H124 to Q95, highlighting the position of three structural waters. The first two mediate interactions between residues H124 and Y156, and the third establishes an H-bond interaction with the ß-hydroxyl moiety of cholesterol. Let us note that the presence of water molecules is not uncommon in cholesterol-binding sites,^49^ and the homologous NPC1-NTD contains two crystallographic water molecules that overlap with the water wire positions (Figure S7).

### Epi-Cholesterol Binds Using Two Binding Modes in the NPC1L1-NTD Domain

To understand the molecular basis of the inability of epi-cholesterol to effectively compete with cholesterol for binding to NPC1L1-NTD, four independent unbiased MD simulations of the epi-cholesterol-bound complex were performed. We used the crystallographic binding mode of cholesterol to NPC1-NTD as a template to model the starting binding mode of epi-cholesterol in the NPC1L1-NTD complexes.

**Figure 3:**
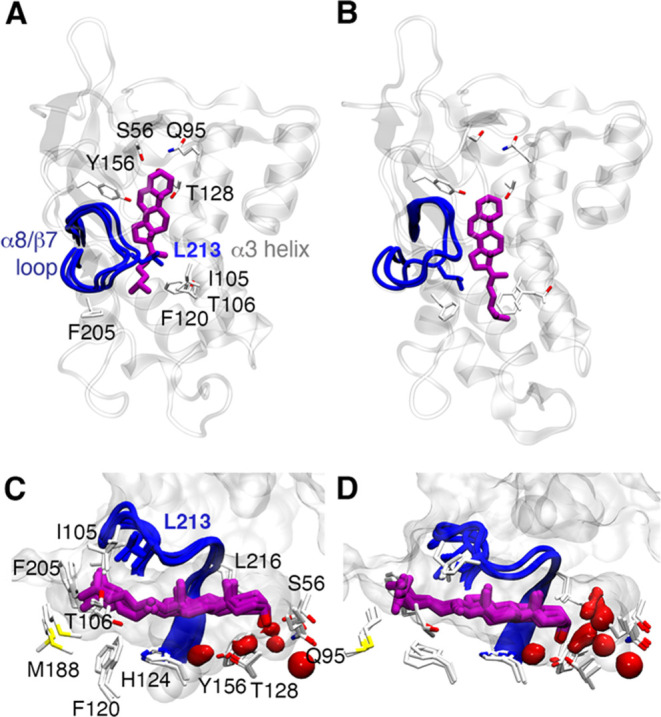
Superposition of the final structures of the MD trajectories of the Q95 (A, C) and T128 binding modes (B, D) of the epi-cholesterol-bound states. (A, B) The conformations sampled by the α8/β7 loop are shown in a blue cartoon. Epi-cholesterol is shown in purple sticks. (C, D) Shape and solvation pattern of the binding site of NPC1L1-NTD for the epi-cholesterol-bound states. The protein surface is shown as a gray surface. The red isosurface accounts for 50% of water occupancy in the binding site during the last 100 ns of each replicate (accumulated 200 ns of simulation time).

The analysis of the trajectories showed that epi-cholesterol displays two distinct binding modes (Figure 3; see also Figure S10). In the first one, epi-cholesterol tries to adopt the Q95 binding mode of cholesterol. However, to keep the H-bond with the side chain of Q95, the orientation of the hydroxyl group forces the sterol core to move closer to the loops with a concomitant displacement of the crystallographic water (Figure 3A,C). In the second, epi-cholesterol is shifted toward the S-opening to establish a H-bond interaction with the side chain of T128 (Figure 3B,D; average distance of 2.8 ± 0.1 Å; present in 72% of the simulation time). We have named this alternative pose as the T128 binding mode. Binding to T128 enlarges the exposure of the aliphatic chain of epi-cholesterol to the bulk solvent, although this effect is counterbalanced by the interaction of the aliphatic side chain with M188 (6.4 ± 1.9% SASA of epi-cholesterol compared to the 3.8 ± 0.6% SASA of epi-cholesterol in the Q95 binding mode). Furthermore, the shift of epi-cholesterol to the T128 binding mode causes the side chain of L213 to move away from I105 while it becomes closer to F205. Therefore, the α8/β7 loop opens slightly when epi-cholesterol binds to T128 compared to the Q95 binding mode, leading to a slight increase of the cavity volume (1153.2 ± 30.9 and 1095.0 ± 7.7 Å^3^, respectively) and filling the cavity with a higher number of water molecules (9 ± 1 and 7 ± 1 water molecules; Figure S11).

It is worth noting that the binding of epi-cholesterol to the Q95 and T128 binding sites exhibits different traits between the four independent replicas. Thus, epi-cholesterol remains primarily bound to T128 in replicas 1 and 3, whereas the occupancy of the Q95 binding site is larger in replicas 2 and 4. Furthermore, differences are also observed in the number of exchanges between the two binding sites, as measured from the difference between the distances of the H-bond interactions formed by epi-cholesterol with Q95 and T128 in conjunction with a threshold of 1.5 Å to split the two binding modes. This analysis revealed that the transitions of epi-cholesterol between Q95 and T128 sites varied from ∼30 exchanges in replica 2 to almost 560 exchanges in replica 3. These differences seem to arise from the behavior of the α8β7 loop, which must open slightly to facilitate the transitions of epi-cholesterol in the interior of the cavity (see above and Figure 3). Therefore, the conformational plasticity of this loop appears to be a relevant factor in assisting the binding of the ligands to NPC1L1-NTD.

**Figure 4:**
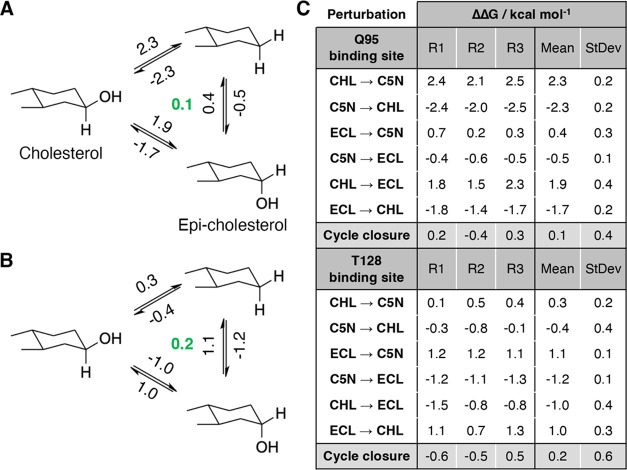
(A, B) Perturbation network of the chemical changes on position 3 of the cholesterol ring for the (A) Q95 binding mode and (B) T128 binding mode. Arrows indicate alchemical simulations carried out with computed relative free energies in kcal mol^–1^. Green numbers indicate average cycle closure errors. (C) Relative free energy changes for the three replicates and the mean and standard deviation of the ΔΔ*G* for each transformation. CHL: cholesterol, ECL: epi-cholesterol, and C5N: cholestane.

On the other hand, these results point out that the sampling obtained from the four independent MD simulations is not complete, and a proper quantitative analysis of the preferences for binding to the Q95 and T128 sites should be gained by using enhanced sampling techniques. In this context, RBFE calculations^50,51^ were first carried out to obtain a quantitative estimate of the change in binding affinity due to epimerization of the hydroxyl group in cholesterol. To this end, alchemical transformations were performed for the conversion of cholesterol and epi-cholesterol to cholestane, which lacks the hydroxyl group and can therefore be used as a reference to estimate the contribution of the H-bond to the binding of cholesterol and epi-cholesterol to the Q95 and T128 binding sites. The simulation systems included the key water molecule that assists the binding of sterols in the Q95 binding mode.^52,53^ The relative free energy values are obtained from the average values of three independent runs (Figure 4).

The results point out that the H-bond formed by cholesterol with Q95 has a net stabilizing contribution of 2.3 kcal·mol^–1^ relative to cholestane, whereas the contribution of the hydroxyl group in epi-cholesterol is around 5-fold less favorable. Indeed, cholesterol binds more favorably to the Q95 binding site than epi-cholesterol by 1.8 kcal·mol^–1^, thus reflecting the inability of the α-configuration of the hydroxyl group to form an effective H-bond in the Q95 binding mode. On the contrary, epi-cholesterol binds more favorably than cholesterol by 1.0 kcal·mol^–1^ in the T128 binding mode, reflecting the formation of the H-bond with T128, which is unfeasible for the β-configuration of the hydroxyl group. In fact, the difference in binding free energy between cholesterol and cholestane is ca. 0.3 kcal·mol^–1^.

**Figure 5:**
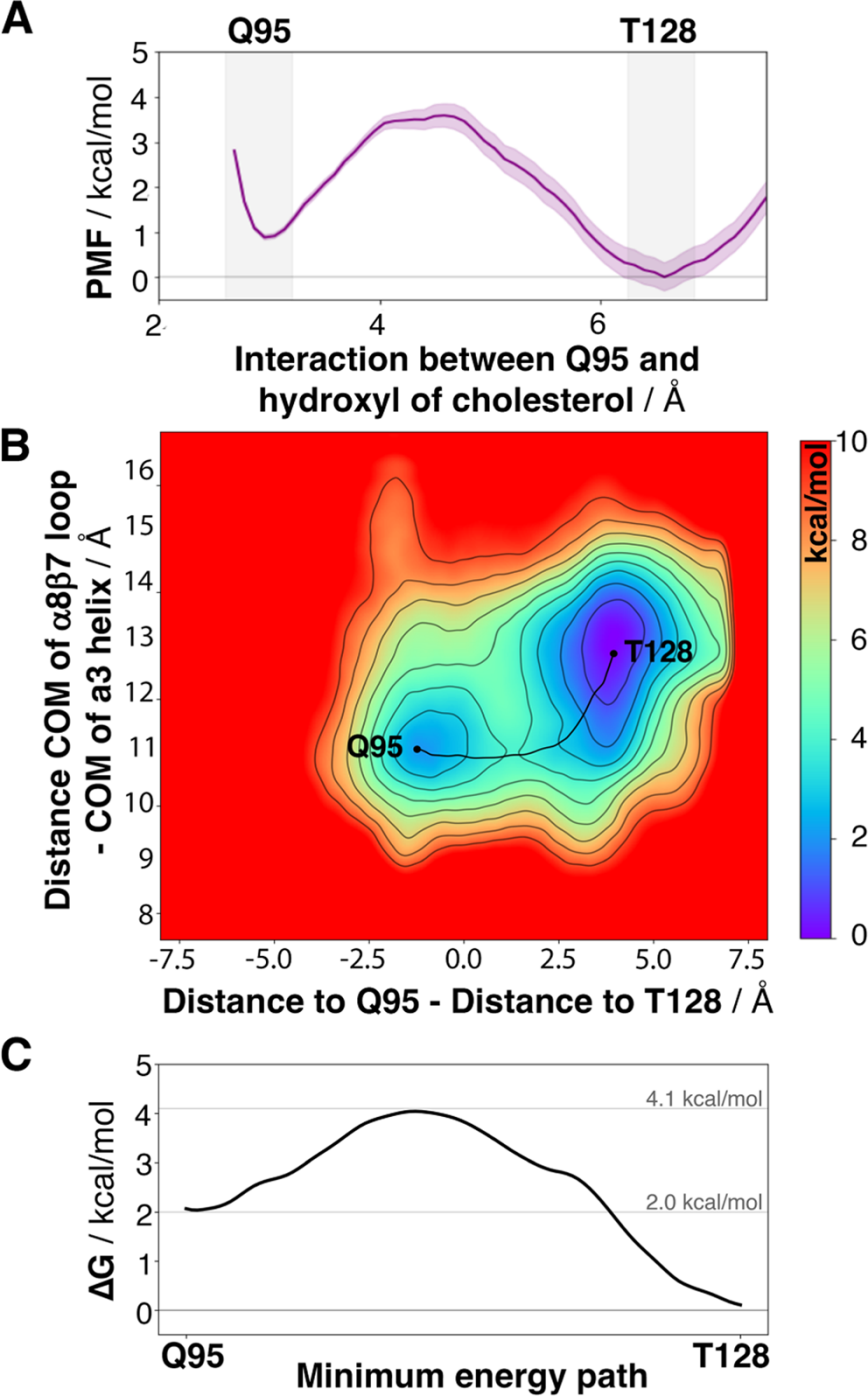
(A) PMF of the interconversion between the Q95 and T128 binding modes obtained by US is in kcal mol^–1^. (B) Free energy surface of the transition between the Q95 and T128 binding modes for epi-cholesterol obtained via two-dimensional (2D)-well-tempered metadynamics. The distances that account for the formation of respective H-bonds between Q95 and T128 with the hydroxyl of epi-cholesterol. The minimum energy path between Q95 and T128 is shown as a black line. The color bar is in kcal mol^–1^. (C) The energetic profile of the minimum energy path between binding modes sampled from the bidimensional free energy surface in kcal mol^–1^.

These results provide a rationale for the stability of the binding mode observed for cholesterol in the unbiased MD simulations, which showed that the ligand remains tightly bound to Q95, in contrast to the transition observed for epi-cholesterol to the T128 binding mode in two trajectories. Nevertheless, keeping in mind the limited sampling attained from the four independent unbiased simulations, we employed two enhanced sampling techniques to estimate the energetic cost of the transition: one-dimensional umbrella sampling calculations (US) using only the formation of the H-bond with Q95 side, and bidimensional well-tempered metadynamics simulations that also consider the opening of the α8β7 loop. First, the potential of mean force (PMF) determined from US calculations reveals the presence of two minima at 2.9 and 6.5 Å, which correspond to the Q95 and T128 binding modes, respectively. The Q95 binding mode is destabilized by approximately 1.1 kcal mol^–1^ with respect to the global minima at the T128 binding mode. The energetic barrier for the transition from the T128 binding site to the Q95 one is 3.6 kcal mol^–1^, whereas the barrier for the Q95 →T128 transition is roughly 2.5 kcal mol^–1^. These energetic barriers were refined using well-tempered metadynamics simulations, which exploit the difference of distances between the O atom of epi-cholesterol and the Q95 and T128 side chains (CV1) and the opening of the α8β7 loop (CV2), defined as the distance between the center-of-mass (COM) of residues 212–214 within the α8β7 loop and residues 104–106 located at the α3 helix.^54^ The resulting FES (Figure 5) shows two minima that correspond to the Q95 and T128 binding modes. The global minimum located at the T128 binding mode is more stable than the Q95 binding mode by ca. 2.0 kcal mol^–1^. While the Q95 binding mode shows a preference for closed conformations of the loop, the T128 binding site can accommodate a larger range of loop conformations. The minimum energy path between the two minima obtained by the String method^45^ is shown in a black line on top of the FES (Figure 5). The barrier for the passage from Q95 to T128 amounts to ca. 2.1 kcal mol^–1^, whereas the barrier for the T128 →Q95 transition is close to 4.1 kcal mol^–1^. The energetic profiles obtained by the well-tempered metadynamics simulations and US calculations are qualitatively similar. However, it is also clear that the motions of the S-entrance may have an impact on the quantitative features of the FES, thus highlighting the coupling between the fluctuations between the two H-bond states of epi-cholesterol and the conformational flexibility of the α8β7 loop and the α3 helix. Overall, these results support the existence of distinct binding modes for cholesterol and epi-cholesterol, revealing that epimerization of cholesterol weakens the binding affinity by promoting the binding to a less buried site in the cavity (Figures 4 and 5).

### Binding Affinity of 25-Hydroxycholesterol and Lanosterol to NP1CL1-NTD

To further explore these findings, an additional set of four independent MD simulations was performed for the complexes of NPC1L1-NTD with 25-hydroxycholesterol and lanosterol (Figure 5) since ^3^H-cholesterol competitive binding assays have shown that these compounds can displace cholesterol from the NPC1L1-NTD binding site.^15,18^ Following the previous discussion, the analysis was focused on the effect of the chemical differences present in these compounds relative to cholesterol (i.e., the location or the double bond and the presence of methyl groups in the tetracyclic core and the hydroxyl or double bond introduced in the aliphatic chain) on the binding to NPC1L1-NTD.

The RMSD of the protein backbone was stable in all cases, with values around 2 Å for the two sterol-bound complexes (see Figures S13 and S14). Compared to cholesterol, which is stably bound (RMSD close to 1 Å), slightly larger RMSD values, generally lower than 3 Å, were observed for the ligand in the complexes with NPC1L1-NTD, which in most cases reflects fluctuations of the aliphatic side chain of the sterols outside the binding pocket.

**Figure 6:**
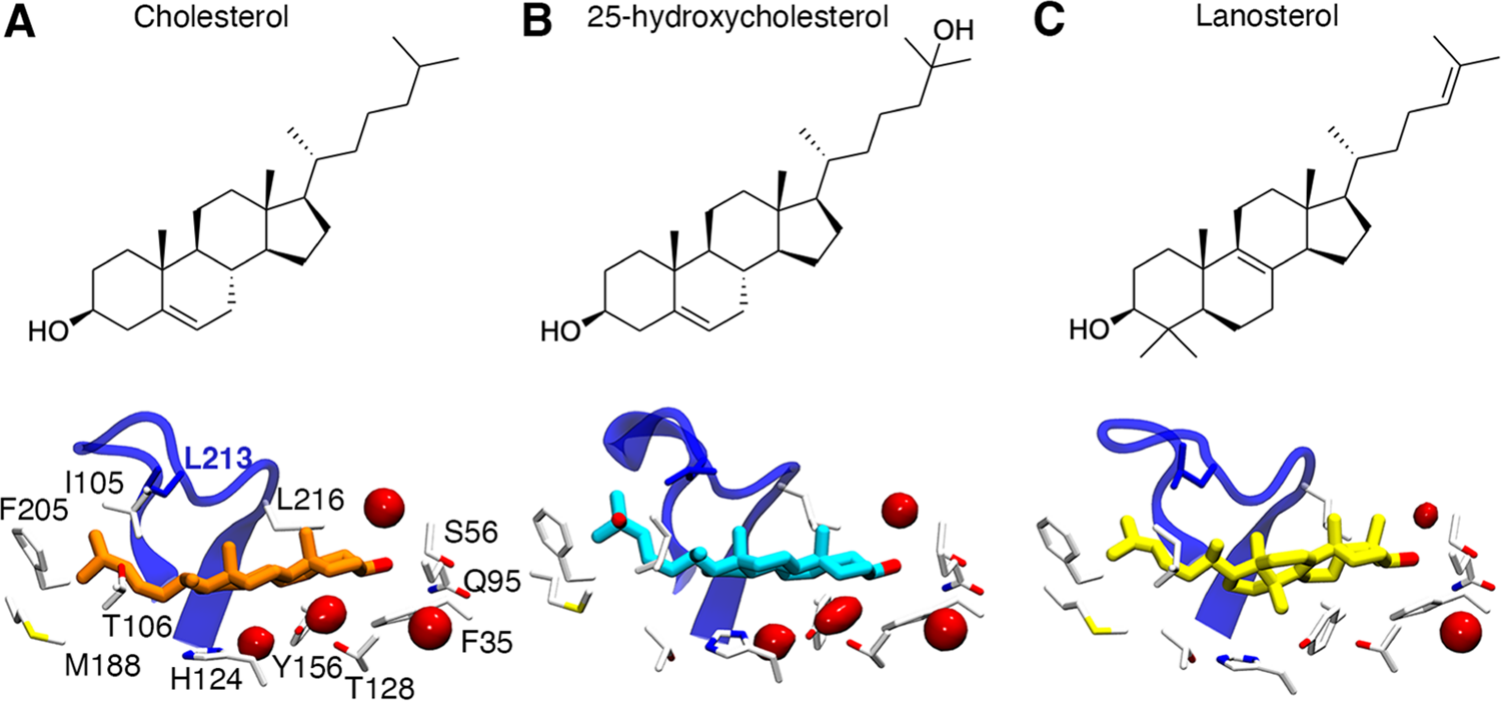
Chemical structures and representative structures of the binding modes of 25-hydroxy-cholesterol and lanosterol bound to the NPC1L1-NTD. Sterols are shown as colored sticks: cholesterol (A, orange), 25-hydroxycholesterol (B, blue), and lanosterol (C, yellow). NPC1L1-NTD is shown as a white cartoon. The position of the α8/β7 loop is shown as a blue cartoon. The occupancy of water molecules in the binding site over 50% of the simulation time is shown as a red isosurface.

The chemical modifications introduced in these compounds do not alter the Q95 binding mode of the sterols (Figure 6). The binding mode of 25-hydroxycholesterol retains all of the described interactions for cholesterol, especially regarding the H-bond with Q95. On the other hand, the presence of the additional hydroxyl group bound at the C25 position can establish additional interactions with the solvent, the side chain of T106 (average distance of 2.8 ± 0.1 Å; present in 25% of the simulation time), the backbone of S102 (average distance of 2.8 ± 0.1 Å; 17% of the simulation time), and the side chain of Q206 (average distance of 2.9 ± 0.1 Å; 8% of the simulation time) (Figure S13 and Table S3).

Despite the chemical changes introduced in the core of the sterol moiety, lanosterol establishes the same interactions described for cholesterol in the Q95 binding mode (Figure 6). The additional methyl groups in the C4 position fit well into the pocket shaped by residues L216, Y156, and F35 and do not impair the formation of H-bonds with Q95 and S56 (Figure S14 and Table S3). Thus, an average distance of 2.9 ± 0.1 Å was determined for the H-bond interaction with Q95 (present in 70% of the simulation time), whereas the interaction with the S56 side chain (average distance of 2.8 ± 0.1 Å) is found in 13% of the simulation time.

The number of water molecules found in the binding site is similar for all of the sterols in the Q95 binding mode, as it amounts on average to 5 ± 1, 6 ± 1, and 4 ± 1 for cholesterol, 25-hydroxycholesterol, and lanosterol, respectively (Figures 6 and S15). In the latter case, the lower content of water molecules can be attributed to the disruption of the water wire caused by the two methyl groups bound at the C4 position. In all sterol complexes, the hydroxyl moiety of the sterol core establishes an H-bond interaction with a water molecule, which, as mentioned before, occupies the same position found in the crystallographic structure of the cholesterol–NPC1 complex (Figure S7).

Finally, MM-PBSA calculations were performed to examine the contribution of the residues in the binding pocket as well as of the water molecule to the binding affinity and selectivity of cholesterol, epi-cholesterol, 25-hydroxycholesterol, and lanosterol (Table 1). Including the water molecule, the binding affinity of cholesterol is estimated to be −20.8 ± 0.9 kcal·mol^–1^, which is close to the values obtained for 25-hydroxycholesterol and lanosterol (−19.3 ± 2.6 and −19.2 ± 2.3 and, respectively). This agrees with the experimental evidence,^7,15,18^ which shows that both 25-hydroxycholesterol and lanosterol exhibit a direct competition with cholesterol for the binding to NPC1L1-NTD. Notably, the absence of the water molecule reduces the binding affinity to 15–16 kcal·mol^–1^. In contrast, the binding affinity estimated for the two binding modes of epi-cholesterol is −15.6 ± 2.2 (Q95 binding site) and −19.2 ± 0.2 kcal·mol^–1^ (T128 binding site). Although caution is required to not overemphasize the significance of the differences in the calculated binding affinities, which nevertheless benefit from the cancellation of uncertainties due to the comparison between structurally related sterols, these results point out that the suboptimal geometry of the C3 position to establish H-bonds and the loss of the interaction with the water molecule destabilizes the binding of epi-cholesterol to the Q95 binding site.

**Table 1:** Binding Free Energies [Average and Standard Deviation (SD); kcal·mol^–1^] Obtained from MM-PBSA Calculations for the Studied Sterols in Complex with NPC1L1-NTD

	MM-PBSA without water molecule			MM-PBSA with water molecule		
	mean	SD	standard error of the mean	mean	SD	standard error of the mean
cholesterol	–16.0	0.6	0.3	–20.8	0.9	0.5
epi-cholesterol Q95 binding mode	–15.6	2.2	1.5			
epi-cholesterol T128 binding mode	–19.2	0.2	0.2			
25-hydroxy-cholesterol	–15.0	1.5	0.7	–19.3	2.6	1.3
lanosterol	–15.0	2.4	1.2	–19.2	2.3	1.1

A per-residue decomposition of the binding affinity provided a consistent view of the most relevant residues for the binding of sterols to NPC1L1-NTD (Table 2). The key residues involved in the H-bond, namely, Q95 and S56, contribute around −3.4 ± 0.5 and −1.6 ± 0.9 kcal·mol^–1^, respectively. The interaction with T128 is only relevant for epi-cholesterol in the T128 binding mode (−2.1 ± 0.1 kcal·mol^–1^), whereas this residue has a negligible effect for the other sterols (≥−0.5 kcal·mol^–1^). Other polar residues are common to both binding modes, such as H124, with an average value of −1.1 ± 0.9 kcal·mol^–1^. The hydrophobic contacts between the tetracyclic core of the sterols and the binding site include L99 (−1.9 ± 0.3 kcal·mol^–1^), L103 (−1.2 ± 0.2 kcal·mol^–1^), and F120 (−0.6 ± 0.3 kcal·mol^–1^), and the α8/β7 loop residues L213 (−1.6 ± 0.4 kcal·mol^–1^), A214 (−1.0 ± 0.2 kcal·mol^–1^), P215 (−1.6 ± 0.3 kcal·mol^–1^), and L216 (−0.8 ± 0.2 kcal·mol^–1^) (see Table 2).

**Table 2:** Per-Residue Decomposition to the Binding Affinity (kcal·mol^–1^) Obtained from MM-PBSA for All of the Studied Sterols in Complex with NPC1L1-NTD^a^

residue	cholesterol	epi-cholesterol Q95 binding mode	epi-cholesterol T128 binding mode	25-hydroxy-cholesterol	lanosterol
E38	–0.6 ± 0.4	0.5 ± 0.6	–0.4 ± 0.2	–0.6 ± 0.3	–0.5 ± 0.5
N54	–0.5 ± 0.3	0.1 ± 0.4	–0.1 ± 0.1	–0.4 ± 0.3	–0.2 ± 0.3
S56	–1.6 ± 0.9	–0.7 ± 0.3	–0.0 ± 0.0	–1.4 ± 0.8	–0.7 ± 0.2
**T61**	**0.0 ± 0.0**	**0.0 ± 0.0**	**0.0 ± 0.0**	**0.0 ± 0.0**	**0.0 ± 0.0**
K94	–0.2 ± 0.1	–0.2 ± 0.1	0.1 ± 0.1	–0.2 ± 0.1	–0.1 ± 0.1
Q95	–3.4 ± 0.5	–3.2 ± 0.5	0.0 ± 0.2	–3.5 ± 0.5	–3.6 ± 0.5
S98	–0.5 ± 0.3	–0.7 ± 0.4	–0.1 ± 0.1	–0.6 ± 0.3	–0.6 ± 0.3
L99	–1.9 ± 0.3	–2.0 ± 0.3	–1.4 ± 0.3	–1.9 ± 0.3	–2.2 ± 0.3
L103	–1.2 ± 0.2	–1.2 ± 0.2	–1.6 ± 0.3	–1.0 ± 0.2	–1.1 ± 0.3
**I105**	–0.2 ± 0.1	**-**0.2 ± 0.1	–1.1 ± 0.3	–0.4 ± 0.2	–0.3 ± 0.2
**L110**	**0.0 ± 0.0**	**0.0 ± 0.0**	–0.2 ± 0.1	**0.0 ± 0.0**	**0.0 ± 0.0**
F120	–0.6 ± 0.3	–0.6 ± 0.2	–1.0 ± 0.2	–0.6 ± 0.2	–0.6 ± 0.2
H124	–1.3 ± 0.4	–1.0 ± 0.4	–1.2 ± 0.5	–1.0 ± 0.3	–0.6 ± 0.5
**T128**	–0.3 ± 0.3	–0.5 ± 0.4	–2.1 ± 0.1	–0.3 ± 0.2	–0.3 ± 0.2
**N132**	**0.0 ± 0.0**	**0.0 ± 0.0**	**0.0 ± 0.0**	**0.0 ± 0.0**	**0.0 ± 0.0**
**F205**	–0.2 ± 0.3	–0.2 ± 0.3	–0.6 ± 0.2	–0.3 ± 0.3	–0.3 ± 0.2
L213	–1.6 ± 0.4	–1.0 ± 0.3	–1.5 ± 0.3	–1.6 ± 0.4	–1.5 ± 0.4
A214	–1.0 ± 0.2	–0.8 ± 0.2	–1.0 ± 0.2	–0.9 ± 0.3	–0.9 ± 0.2
**P215**	–1.6 ± 0.3	–1.5 ± 0.3	–3.2 ± 0.1	–1.6 ± 0.3	–1.2 ± 0.3
**L216**	–0.8 ± 0.2	–0.9 ± 0.2	–1.6 ± 0.3	–0.7 ± 0.2	–1.1 ± 0.2
water	–1.6 ± 0.6			–1.7 ± 0.5	–1.3 ± 0.6

a Residues highlighted in gray are key positions for cholesterol absorption identified in the literature.

### Mutations that Impair Cholesterol Transport Distort the α8/β7 Loop

Previous studies have disclosed a variety of single-point mutations in the NPC1L1-NTD that have a detrimental effect on cholesterol absorption.^18,55,56^ In light of the previous results regarding the binding mode of cholesterol, a discussion of the potential impact of mutations that affect specific residues in the binding pocket of NPC1L1-NTD deserves interest. For our purposes, the T128A, I105A, F205A, and P215A variants, which trigger a reduction in cholesterol absorption varying from 35 to 85% (Table 3),^18^ were selected to explore their effect on cholesterol binding. Noteworthily, although the detrimental effect of P215A may be justified by the loss of the contribution of P215 to the binding affinity, which varies from −1.6 to −1.2 kcal·mol^–1^ for cholesterol, 25-hydroxycholesterol, and lanosterol (see Table 2), the effect of the other three variants is not apparent, as their contribution to the binding affinity is around −0.3 kcal·mol^–1^ for these compounds. In turn, this suggests that other factors beyond the direct interaction between the ligand and the residues in the binding pocket may influence the efficiency of binding of cholesterol to NPC1L1-NTD.

**Table 3:** Binding Free Energies (Average and Standard Deviation (SD); kcal·mol^–1^) Obtained Using MM-PBSA Calculations for Cholesterol Bound to the Mutated Variants of NPC1L1-NTD

	reduction of cholesterol absorption %	MM-PBSA with water molecule kcal mol^–1^		
		mean	SD	standard error of the mean
wild-type	−	–20.8	0.9	0.5
**I105A**	35	–22.1	1.9	1.0
**T128A**	85	–18.1	2.7	1.4
**F205A**	85	–19.8	1.5	0.7
**P215A**	52	–15.7	2.2	1.1

To gain insight into this question, four independent MD simulations were performed for both the apo and cholesterol-bound states of the mutated (T128A, I105A, F205A, and P215A) NPC1L1-NTD and a comparison was made with the structural and energetic features determined for the wild-type protein (see above). The RMSD profile of the protein backbone of the mutated variants showed no significant alterations for both the apo and cholesterol-bound simulations (RMSD values around 2 Å; see Figures S16–S19 and S20–S23 for apo and ligand-bound species, respectively). The RMSD values of cholesterol for all of the mutants were close to 1 Å and, therefore, comparable to the cholesterol-bound complex formed with the wild-type NPC1L1-NTD (Figure S4), with the only exception of a few transient peaks, always below 3 Å, which account for rearrangements of the aliphatic side chain.

**Figure 7:**
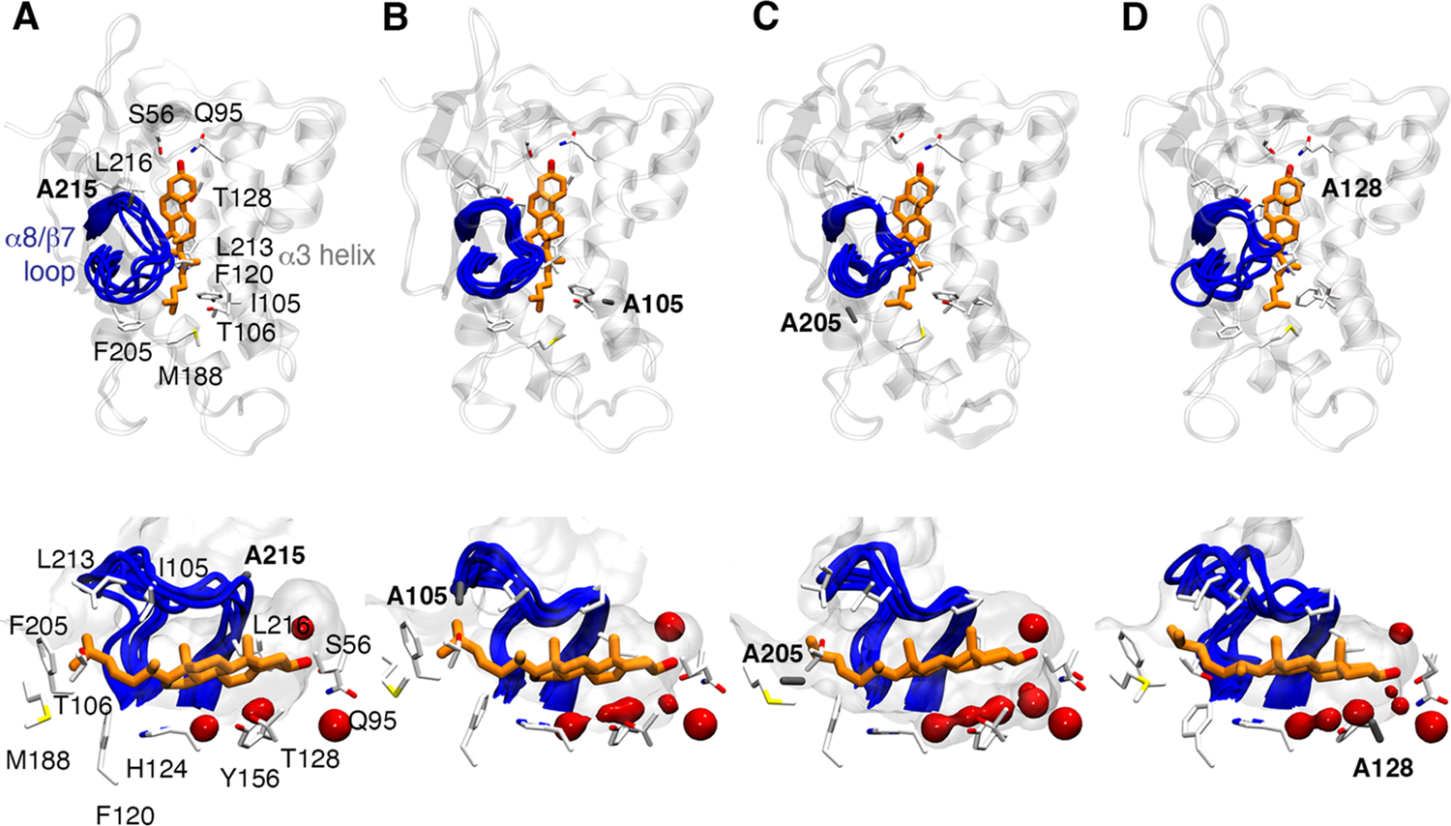
(Top) Q95 binding mode of cholesterol is maintained in the (A) P215A, (B) I105A, (C) F205A, and (D) T128A mutated variants of NPC1L1-NTD. (Bottom) Shape and solvation pattern of the binding site of NPC1L1-NTD for cholesterol-bound states. The conformations sampled by the α8/β7 loop are shown in a blue cartoon. Cholesterol molecules are shown as orange sticks. Mutated residues are highlighted using gray sticks. The protein surface is shown as a gray surface. The red isosurface accounts for 50% of water occupancy in the binding site during the last 100 ns of each replicate (accumulated 400 ns of simulation time).

Compared to the wild-type complex, there is a slight reduction in the volume of the cavity, which varies from 940 to 990 Å^3^ for the mutated variants (volume of 1024 ± 19 Å^3^ for the wild-type protein). Nevertheless, the Q95 binding mode is stable along the simulation time in all of the cholesterol-bound simulations (Figures 7 and S20–S23). Indeed, the three key H-bond interactions with Q95, S56, and water molecules are maintained in all replicates. We only observe two short-lived destabilizations of the Q95 binding mode in the T128A mutant, where cholesterol moves toward the S-opening with a water molecule bridging the interaction with Q95.

The effect of each mutation on the cholesterol binding to NPC1L1-NTD was examined using MM-PBSA calculations (Table 3). In agreement with the trends discussed from the data in Table 2, the P215A mutation gives rise to a significant decrease in the binding affinity of cholesterol (−15.7 ± 2.2 kcal·mol^–1^) compared to the wild-type protein (−20.8 ± 0.9 kcal·mol^–1^), which partially accounts for the loss of the proline side chain contribution of 1.6 ± 0.3 kcal·mol^–1^. Nevertheless, the values obtained for I105A, T128A, and F205A (−19.2 ± 2.3, −19.3 ± 2.6, and −21.0 ± 1.9 kcal·mol^–1^, respectively) are close to the binding affinity estimated for the wild-type complex, suggesting that other factors may affect the binding process.

**Figure 8:**
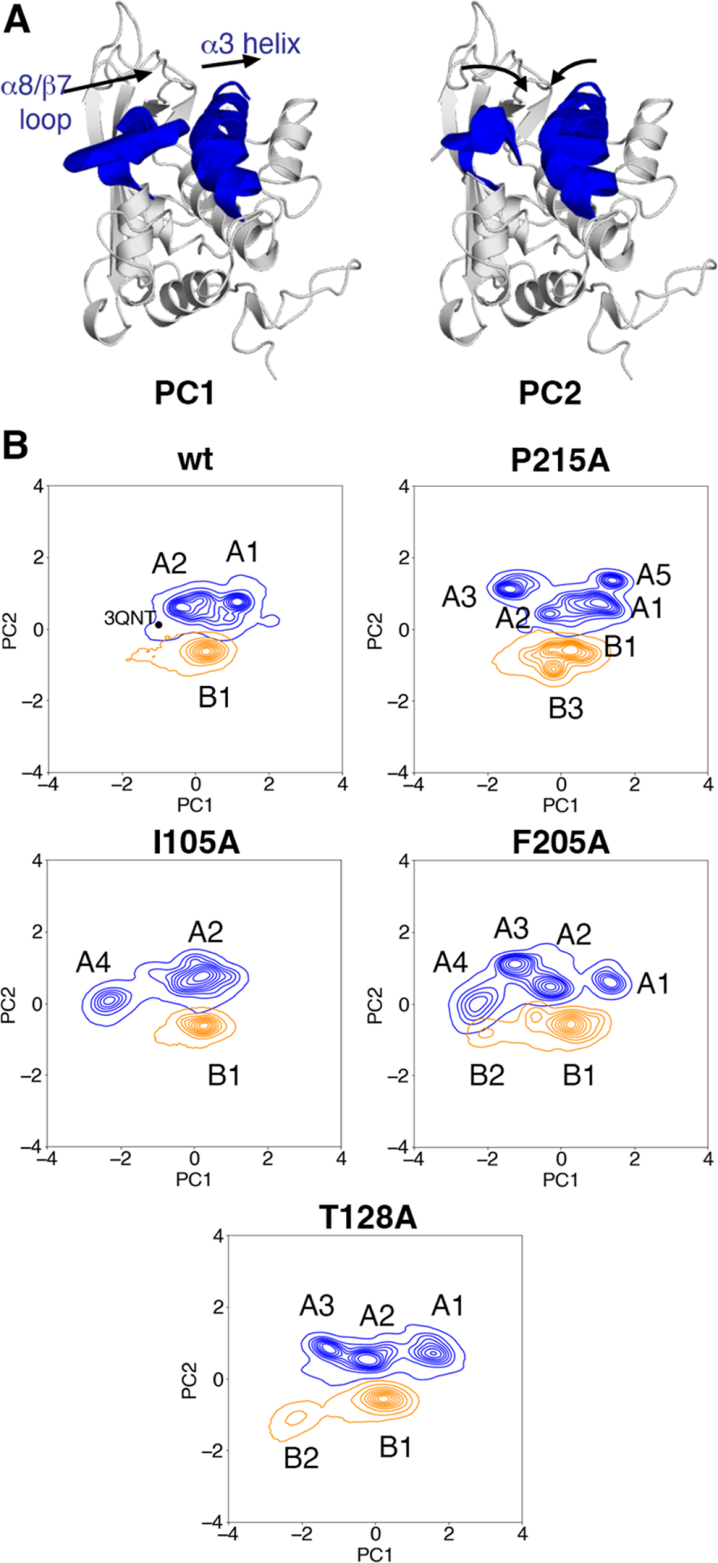
Principal component analysis of the wild-type NPC1L1-NTD domain and its mutated variants. (A) Structural representation of the two main principal components of the PCA. PC1 accounts for the sideways motion between the α8/β7 loop and helix α3, and PC2 accounts for the motion that reduces the distance between the α8/β7 loop and helix α3. (B) Projections of the wild-type protein and the mutated variants on the space defined by the first two Principal Components. The density of visited conformations of the trajectories of the apo and cholesterol-bound are shown as blue and orange-shaded contours, respectively. For the wild-type protein, the location of the X-ray structure of the apo species is also indicated.

Considering these results, principal components analysis (PCA) was used to examine the impact of the mutations on the conformational dynamics of the α8/β7 loop and the α3 helix, which contribute to shaping the binding pocket in the apo and cholesterol-bound species (Figure 8). The two first PCs explain almost 66% of the total structural variance. The first PC (PC1) accounts for 39% of the structural variance and describes a concerted lateral motion of the α8-β7 loop and the α3 helix, which opens the entrance of the binding site. The second PC (PC2) explains 27% of the variance and describes the concerted motion that brings the α8-β7 loop into the binding pocket and closer to the α3 helix, which is the main driver of the narrowing of the binding pocket entrance. The third and the fourth components only explained 8 and 7% of the total variance.

**Figure 9:**
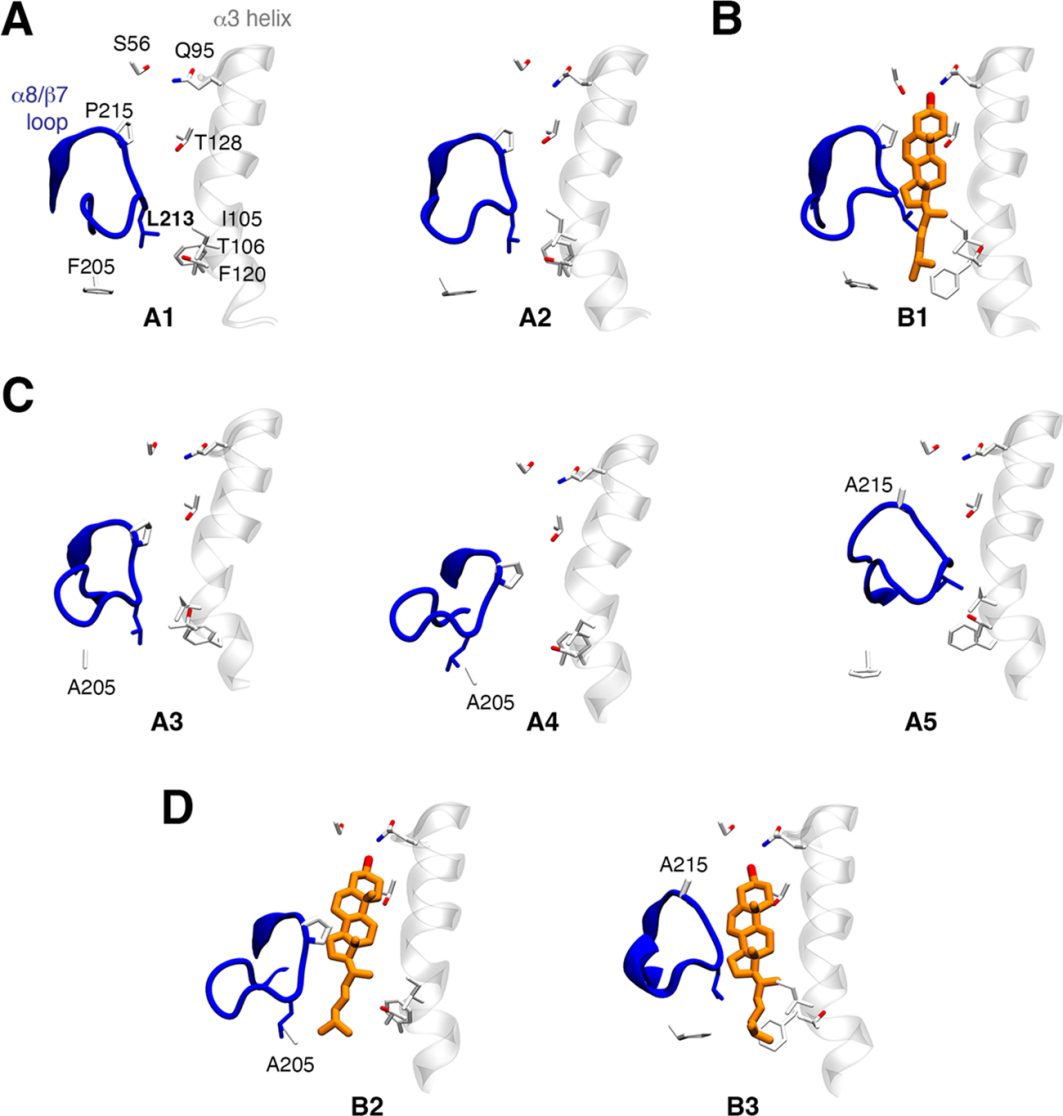
Representative structures of the main conformations of the α8/β7 loop and α3 helix in (A, B) the wild-type and (C, D) the mutated variants of NPC1L1-NTD. A representative snapshot of the α8/β7 loop and α3 helix sampled in (A, C) the apo and (B, D) cholesterol-bound trajectories is shown as blue and white cartoons, respectively. Cholesterol is shown as orange sticks. Representative structures of the alternative conformations A3, A4, and B2 are extracted from the F205A mutant. Representative structures of the conformations A5 and B3 are only present in the P215A variant.

The apo- and cholesterol-bound trajectories sample two distinct populations in the PC space. PC2 discriminates between apo and holo states of the NPC1L1-NTD, as apo and cholesterol-bound trajectories populate positive and negative values along the PC2 projection, respectively (Figure 8). On the other hand, the different conformations of the α8/β7 loop sampled in the simulations spread along positive and negative values of PC1, leading to two clusters for the wild-type apo species (Figure 9; see also Table S3): in one case the α8/β7 loop is buried in the binding site placing the L213 side chain close to F120 (A1: 24 ± 2%, pocket volume of 483 ± 116 Å^3^) and in the other, the L213 backbone forms a H-bond with S102 (3.0 ± 0.7 Å) and a hydrophobic contact with I105A (A2: 75 ± 2%, pocket volume of 491 ± 145 Å^3^). In the holo species, NPC1L1-NTD populates a single conformation (B1: 97 ± 2%, pocket volume of 1025 ± 61 Å^3^).

The P215A variant distorts the dynamics of the α8/β7 loop, leading to four conformations of the entrance of the binding site in the apo species (Figures 8 and 9; see also Table S4). Compared to the wild-type protein, whereas the A1 conformation is not significantly altered (A1: 24 ± 1%, pocket volume of 361 ± 76 Å^3^), the A2 conformation is less populated (A2: 42 ± 1%, pocket volume of 390 ± 55 Å^3^) in favor of two new conformational states. In one of them (A3: 25 ± 2%, pocket volume of 444 ± 65 Å^3^), the α8/β7 loop is stabilized by a H-bond between A214 and S102 (3.6 ± 0.9 Å), which shifts the L213 side chain from the entrance and interacts with F205. In the other (A5: 9 ± 1%, pocket volume of 288 ± 22 Å^3^), the α8/β7 loop binds deeper into the binding site, with L213 being located closer to H124. In the holo state, however, the impact of the P215A mutation is weaker, as the B1 conformation (79 ± 1%, pocket volume of 902 ± 51 Å^3^) is largely populated, and a minor conformation (B2: 20 ± 1%, pocket volume of 940 ± 72 Å^3^), where the α8/β7 loop adopts a slightly tighter conformation around the sterol molecule, retains the Q95 binding mode (Figure 7D). Altogether, the P215A mutation alters primarily the conformational space of the apo species, stabilizing alternative conformations with significantly reduced volume of the binding site and reducing the binding affinity of cholesterol in the holo species (see Table 3). Altogether, these results provide a structural basis to explain the experimental reduction (48%) of cholesterol absorption.^18,56^

Mutations on residues I105 and F205, which stabilize the hydrophobic cluster found in the closed state of the apo state, are likely to alter the conformational preferences of the α8/β7 loop. With regard to the I105A variant, although the conformation A2 is the most populated (76 ± 5%) and resembles the cluster found in the wild-type apo species, there is a marked reduction in the pocket volume (264 ± 43 Å^3^). Furthermore, the A1 conformation disappears, and a minor conformational state (A4: 18 ± 3%, pocket volume of 525 ± 44 Å^3^) emerges (Figures 8 and 9; see also Table S4). This reflects the weaker stability of the hydrophobic cluster resulting from the Ile → Ala mutation, which favors the opening of the binding site and the enlargement of the cavity. In contrast, the I105A mutation does not significantly alter the conformation dynamics of the cholesterol-bound state (B1: 93 ± 1%, pocket volume of 985 ± 52 Å^3^). These results are in qualitative agreement with the computed binding affinity of cholesterol for this mutated variant, which compares with the value obtained for the wild type (see Table 3), the mild effect on the cholesterol absorption, as the I105A mutation leads to the lowest reduction (35%) among the four selected variants.^18,56^

Compared to I105A, the F205A variant gives rise to a larger reduction of cholesterol absorption (∼85%).^18,56^ This trait agrees with the larger remodeling of the conformational preferences of the α8/β7 loop observed for this mutation (Figures 8 and 9; see also Table S4). Thus, conformations A2 (35 ± 1.0%, pocket volume of 475 ± 126 Å^3^) and A1 (12 ± 1%, pocket volume of 488 ± 123 Å^3^) are less populated relative to the wild-type apoprotein. Furthermore, the α8/β7 loop populates two novel states: A3 (31 ± 1%, pocket volume of 366 ± 43 Å^3^) and A4 (23 ± 1%, pocket volume of 745 ± 95 Å^3^). Since the side chain of F205 is a key component of the hydrophobic cluster that stabilizes the closed state, the mutation to Ala weakens the stability of the cluster, favoring the adoption of other conformations. In contrast, the F205A effect is much less apparent in the cholesterol-bound state, as the B1 conformation is the main cluster (90 ± 1%, pocket volume of 994 ± 45 Å^3^). Thus, although F205 has a residual contribution to the cholesterol-binding affinity, it is key to stabilizing the conformation of the α8/β7 loop in the apo state.

**Figure 10:**
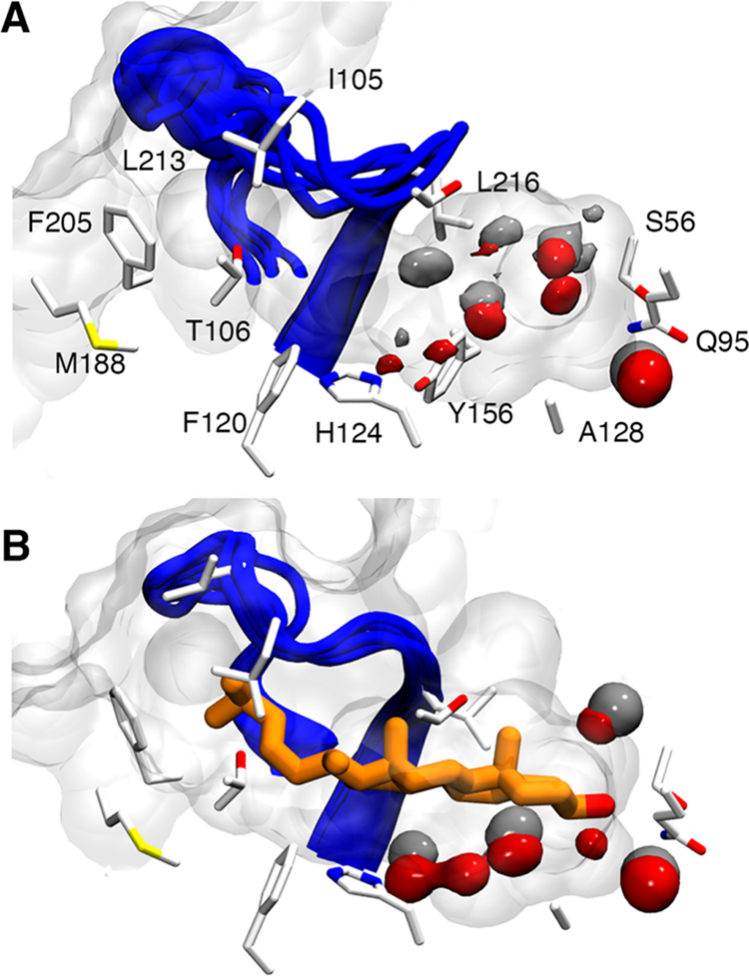
Superposition of the major conformations of (A) the apo and (B) cholesterol-bound species of the T128A variant. The conformations sampled by the α8/β7 loop are shown as a blue cartoon. The protein surface is shown as a gray surface. The isosurface accounts for 50% of water occupancy in the binding site during the last 100 ns of each replicate (accumulated 400 ns of simulation time). The water occupancy for the wild-type protein and T128A mutant are shown as gray and red isocontours, respectively.

The T128A mutant, which also has a drastic impact on cholesterol absorption (reduction of 85%), is more challenging since T128 does not directly interact with the α8/β7 loop. Nevertheless, our results point out that this mutation changes the dynamics of the S-opening, especially in the species. Thus, the main conformation A2 (52 ± 4%, pocket volume of 434 ± 37 Å^3^) is partially shifted to a novel state (A3: 17 ± 3%, pocket volume of 347 ± 86 Å^3^) due to a remodeling of the hydration pattern in the binding site (Figure 10). Compared to the wild-type species, T128A promotes the loss of water located between the backbone of L213 and the side chain of S102, which bridges the interaction between the L213 backbone and the H124 side chain. Of note, in the A1 conformation, the H124 side chain adopts a distinct arrangement that enables this interaction without the water molecule. Upon ligand binding, the S-entrance mainly populates the B1 conformation (86 ± 1%, pocket volume of 935 ± 54 Å^3^), which supports the Q95 binding mode, although a minor conformation (B3: 8 ± 1%, pocket volume of 968 ± 54 Å^3^), which alters the Q95 binding mode by shifting cholesterol toward the S-opening due to a water molecule that bridges the interaction with Q95, is also observed (Figure S24).

**Figure 11:**
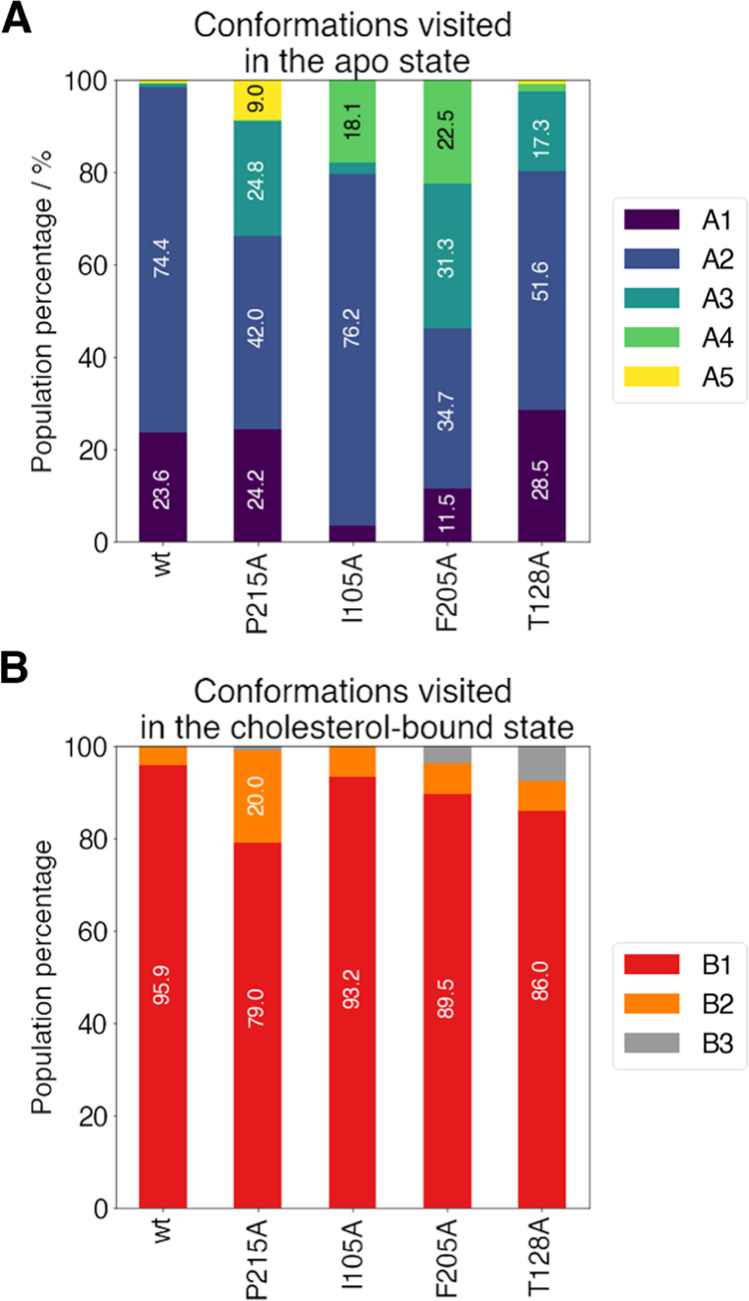
Relative population of each conformation is shown as a stacked bar plot for (A) apo and (B) cholesterol-bound simulations.

Altogether, the analysis of the trajectories sampled for the wild-type NPC1L1-NTD and its mutated variants suggest that the mutations have a minor impact on the conformational preferences of the α8/β7 loop in the cholesterol-bound species, as the main conformation (B1) observed in the wild-type protein is preserved in the mutated proteins (Figure 11). Indeed, the mutations do not substantially alter the binding mode of cholesterol in the Q95 binding site, and in most of the cases, they do not even affect its binding affinity, with the only exception of P215A (Table 3). However, the present results point out that the single-point mutations in key residues with a minor contribution to the binding affinity of cholesterol may have a large impact on the efficiency of cholesterol absorption by altering the conformational landscape of the S-opening in the apo species of NPC1L1-NTD (Figure 11). These findings agree with previous studies that have highlighted the relevant role played by mutations introduced in enzymes to remodel the dynamic equilibrium between the ensemble of differently populated conformational substates, thus strongly modifying not only the catalytic efficiency but also the ability to accommodate alternative substrates.^57−59^ In this context, our results strengthen the role played by the α8/β7 loop, particularly L213 and F205, in modulating the access of cholesterol to the binding pocket of NPC1L1-NTD.^15,17^

## Conclusions

The molecular basis of the epimerization-related selectivity in the binding and transport of cholesterol by the N-terminal domain of the NPC1L1 transporter has been explored by combining molecular dynamics simulations of the apo and holo species, in conjunction with free energy calculations for the wild-type protein and four mutated variants known to cause a detrimental effect on the cholesterol import.

The results have shown the drastic influence of the epimerization of the hydroxyl group on the binding mode of cholesterol and epi-cholesterol. The analysis of the independent MD simulations run for cholesterol consistently supports the stability of the Q95 binding mode, where the hydroxyl moiety establishes H-bond interactions with the side chain of Q95, S56, and a neighboring water molecule. The main features of this binding mode are also stably reproduced for 25-hydroxycholesterol and lanosterol, although additional interactions can be found due to the chemical modifications on the aliphatic tail of 25-hydroxycholesterol and the methyl groups of lanosterol. In contrast, the α-configuration of the C3 atom in epi-cholesterol leads to the T128 binding mode, which is shifted ca. 3 Å to the mouth of the cavity. Indeed, US calculations and well-tempered metadynamics simulations reveal the existence of an energetic barrier (i.e., 4.1 kcal·mol^–1^) for the transition from this binding mode to the Q95 one, thus preventing epi-cholesterol from penetrating deeper in the binding pocket. Altogether, this reflects that the α-configuration of the C3 position is unable to properly bind to the Q95 binding mode and can move to a metastable state of the entrance pathway of sterols.

The binding site of cholesterol is seemingly challenged by the detrimental effect of several single-point mutations in the binding site (T128A, I105A, F205A, and P215A) that impair cholesterol absorption.^18^ Our results showed that these mutations have a mild effect on the conformational space of the α8/β7 loop in the cholesterol-bound state and that only P215A contributes to weaken the binding affinity of cholesterol. In contrast, the results also revealed the impact of these mutations in distorting the conformational landscape of the α8/β7 loop in the apo state, which is key for regulating the access of cholesterol to the binding pocket through the formation of a hydrophobic cluster formed by L213, L103, T106, F120, and F205. The reshaping of the conformational preferences of the α8/β7 loop should destabilize its role in mediating the access of cholesterol to the binding pocket, thus strengthening the hypothesis of the gating mechanism played by the α8/β7 loop.^15,17^

## Data Availability

Gaussian09 (Revision B.01) is a licensed software for electronic structure calculations used in the parametrization of sterol molecules. The Gromacs 2022.4 package is a free, open-source software suite for high-performance molecular dynamics and output analysis. RBFE (Thermodynamic Integration) calculations were performed with the GPU-accelerated implementation of Amber22, and MM-PBSA computations were determined with the MMPBSA.py script available in AmberTools22. Clustering of the ensemble of conformational states was performed using the Robust Perron Cluster Analysis method (PCCA+) as implemented in the PyEMMA 2.0 module.

## Supporting Information Available

Detailed analysis of the independent trajectories run for apo and ligand-bound species of wild-type NPC1L1-NTD and its mutated variants; files with topologies and parameters of cholesterol, epi-cholesterol, 25-hydroxycholesterol, and lanosterol, initial configurations of simulated systems (GRO files) and trajectories are available at Zenodo (PDF)

## Abbreviations

NPC1L1: Niemann–Pick type C1 like 1 transporter
NPC1: Niemann–Pick type C1 transporter
NTD: N-terminal domain
RBFE: relative binding free energies
