# Supplementary material for: Binding of Cholesterol to the N-Terminal Domain of the NPC1L1 Transporter: Analysis of the Epimerization-Related Binding Selectivity and Loop Mutations
Source: J Chem Inf Model. 2023 Dec 28;64(1):189–204. doi: 10.1021/acs.jcim.3c01319 (PMC10777396; doi:10.1021/acs.jcim.3c01319)
Supplement: Supplementary file 1 — ci3c01319_si_001.pdf [file ci3c01319_si_001.pdf]

SUPPORTING INFORMATION

Binding of Cholesterol to the N-terminal Domain of the NPC1L1 Transporter: Analysis of the Epimerisation-Related Binding Selectivity and Loop Mutations

*Aitor Valdivia [1,3], F Javier Luque [1,2,3], Salomé Llabrés [1,3]**

1 Department of Nutrition, Food Sciences and Gastronomy, Campus Torribera, University of Barcelona, Prat de la Riba 171, 08921 Santa Coloma de Gramenet, Spain.

2 Institute of Biomedicine (IBUB), University of Barcelona, 08028 Barcelona, Spain.

3 Institute of Theoretical and Computational Chemistry (IQTC-UB), University of Barcelona, 08028 Barcelona, Spain.

*** e-mail: salome.llabres@ub.edu**

TABLE OF CONTENTS

Figure S1. Structural comparison of the NTD domains of NPC1L1 and NPC1. S4

Figure S2. Superposition of the X-ray structures of the NTD domains of NPC1L1 and NPC1.S5

Figure S3. RMSD profiles of apo NPC1L1-NTD. S6

Figure S4. RMSD profiles of cholesterol-bound NPC1L1-NTD and H-bond interactions of cholesterol over simulation time. S7

Figure S5. Selected interactions of L213 in the apo NPC1L1-NTD over simulation time. S8

Figure S6. Number of water molecules in the binding site of the apo state of NPC1L1-NTD over simulation time. S9

Figure S7. Overlap between the water network of NPC1L1-NTD with the X-ray structures of the NPC1-NTD. S10

Figure S8. Selected interactions of L213 in the cholesterol-bound NPC1L1-NTD over simulation time. S11

Figure S9. Number of water molecules in the binding site of the cholesterol-bound species of NPC1L1-NTD over simulation time. S12

Figure S10. RMSD profiles of epi-cholesterol bound NPC1L1-NTD and H-bond interactions of epi-cholesterol over simulation time. S13

Figure S11. Number of water molecules in the binding site of the epi-cholesterol-bound species of NPC1L1-NTD over simulation time. S14

Figure S12. Representation of the two collective variables along the well-tempered metadynamics over the Collective Variable space. S15

Figure S13. RMSD profiles of 25-hydroxy-cholesterol-bound NPC1L1-NTD and H-bond interactions of 25-hydroxy-cholesterol over simulation time. S15

Figure S14. RMSD profiles of lanosterol-bound NPC1L1-NTD and H-bond interactions of lanosterol over simulation time. S16

Figure S15. Number of water molecules in the binding site of (*left*) 25-hydroxy-cholesterol- and (*right*) lanosterol-bound species of NPC1L1-NTD over simulation time S17

Figure S16. RMSD profiles of apo P215A NPC1L1-NTD over simulation time. S18

Figure S17. RMSD profiles of apo I105A NPC1L1-NTD over simulation time. S19

Figure S18. RMSD profiles of apo F205A NPC1L1-NTD over simulation time. S20

Figure S19. RMSD profiles of apo T128A NPC1L1-NTD over simulation time. S21

Figure S20. RMSD profiles of cholesterol-bound P215A NPC1L1-NTD and H-bond interactions of cholesterol over simulation time. S22

Figure S21. RMSD profiles of cholesterol-bound I105A NPC1L1-NTD and H-bond interactions of cholesterol over simulation time. S23

Figure S22. RMSD profiles of cholesterol-bound F205A NPC1L1-NTD and H-bond interactions of cholesterol over simulation time. S24

Figure S23. RMSD profiles of cholesterol-bound T128A NPC1L1-NTD and H-bond interactions of cholesterol over simulation time. S25

Figure S24. Superposition of the major conformations of (**A,C**) the apo and (**B,D**) cholesterol-bound states of the T128A mutant. S26

Table S1. Available structural information of the NPC1L1 protein on PDB database. S27

Table S2. Available structural information of the NPC1 protein on PDB database. S28

Table S3. Average distance (A) and preservation (%) of the interactions between wild-type NPC1L1-NTD and the studied sterol molecules. S29

Table S4. Description of the main conformational states of the apo and cholesterol-bound systems found for the wildtype NPC1L1-NTD and its mutated variants. S30

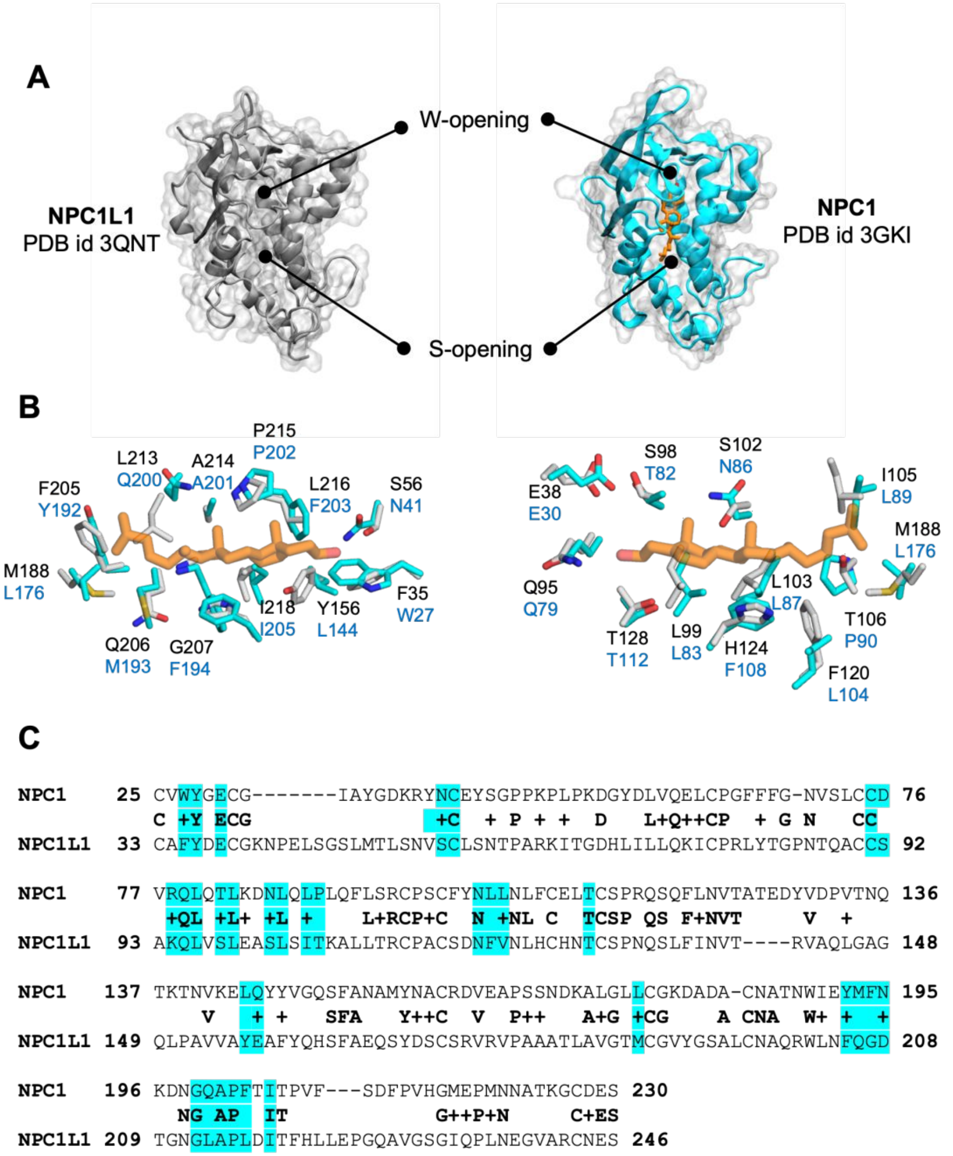

Figure S1. Structural comparison of the NTD domains of NPC1L1 and NPC1. (**A**) The global fold of both NTD domains of NPC1L1 and NPC1 are shown in grey and cyan cartoon respectively. (**B**) Comparison of the residues involved in the cholesterol binding site for both proteins. The crystallographic position of cholesterol in NPC1 is shown in orange sticks. (**C**) Sequence alignment between the NTDs of NPC1L1 and NPC1. Residues in the binding site of cholesterol are highlighted in cyan.

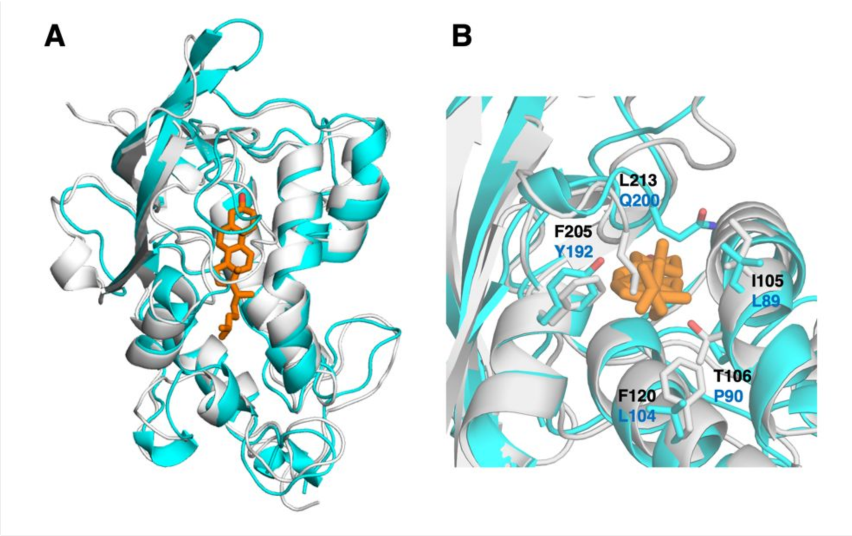

Figure S2. Superposition of the X-ray structures of the NTD domains of NPC1L1 and NPC1. (**A**) The global fold of both NTD domains of NPC1L1 and NPC1 are shown in grey and cyan cartoon respectively. (**B**) Comparison of the residues involved in the entrance of the binding site for both proteins. The crystallographic position of cholesterol in NPC1 is shown in orange sticks.

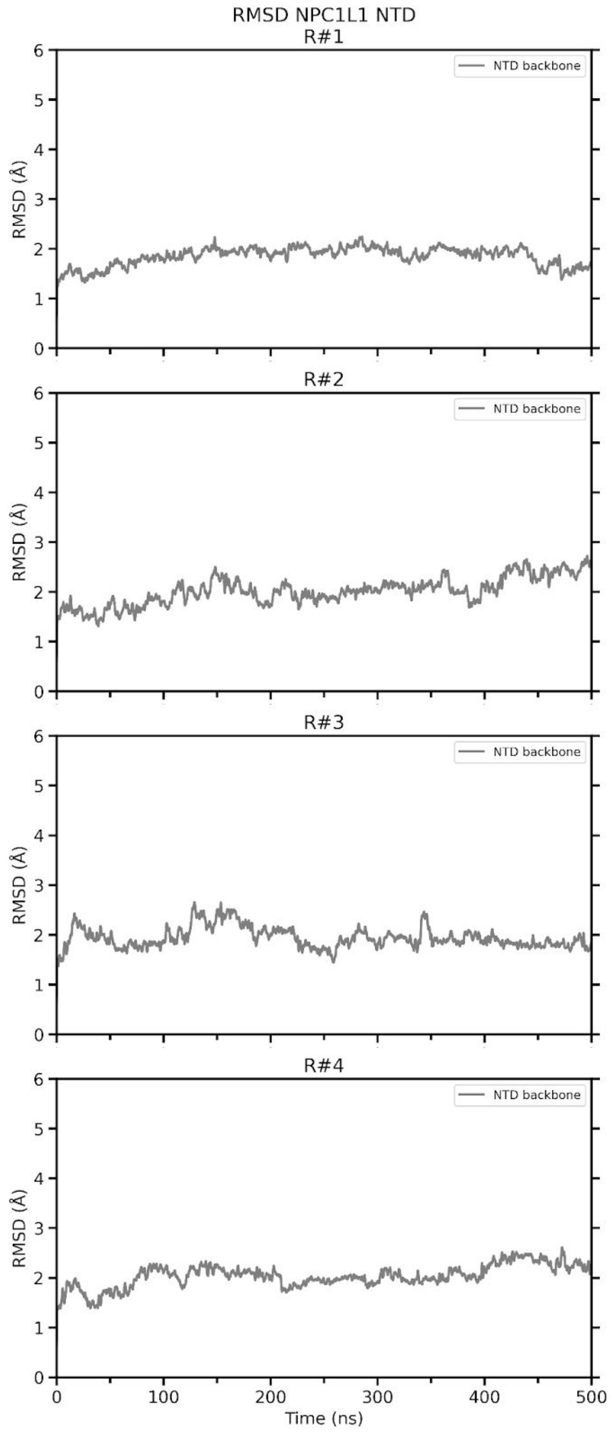

Figure S3. RMSD profiles of the backbone atoms of apo NPC1L1-NTD.

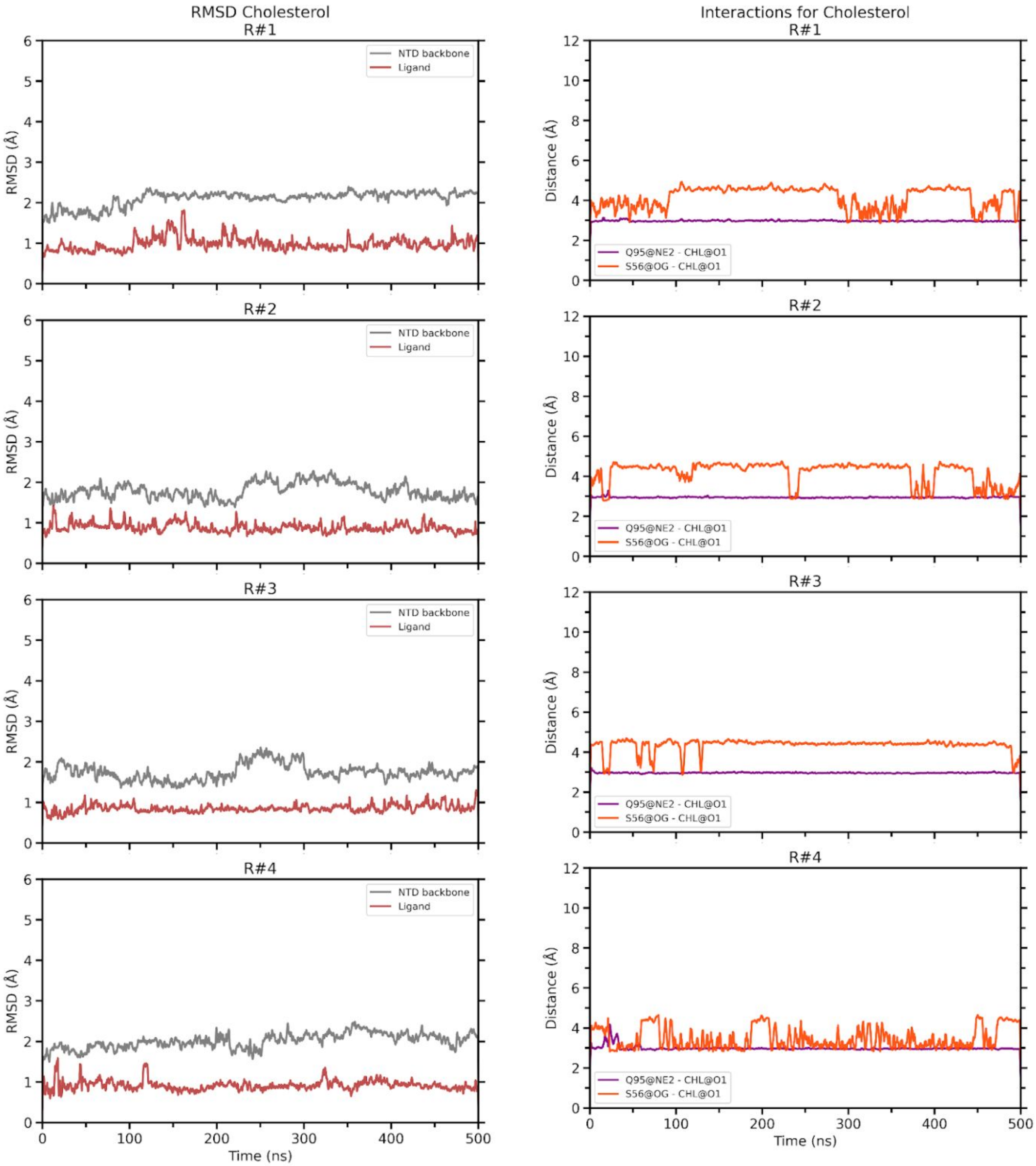

Figure S4. RMSD profiles of the cholesterol-bound NPC1L1-NTD and H-bond interactions of cholesterol over simulation time. (*Left*) Grey and red lines correspond to the RMSD of the backbone of the protein and heavy atoms of the ligand. (*Right*) Purple and orange lines correspond to the H-bonds between the hydroxyl moiety of the ligand and the sidechain of Q95 and S35.

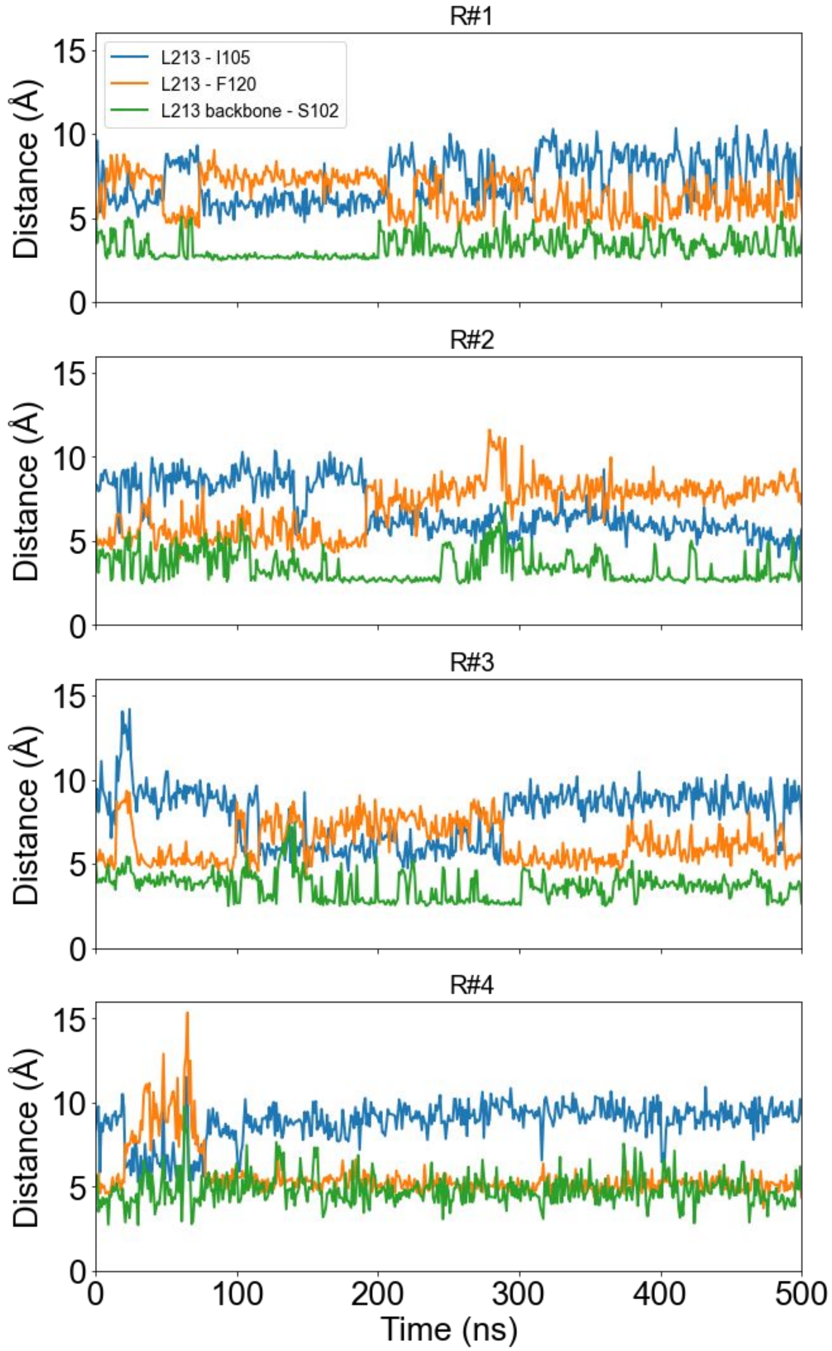

Figure S5. Selected interactions of L213 in the apo NPC1L1-NTD over simulation time. Blue and orange lines correspond to the distances of the CG atom of L213 to the CG atom of I105 and the center of the aromatic ring of F120, respectively. Green lines account for the H-bond interaction between the O atom of the backbone of L213 to the sidechain of S102.

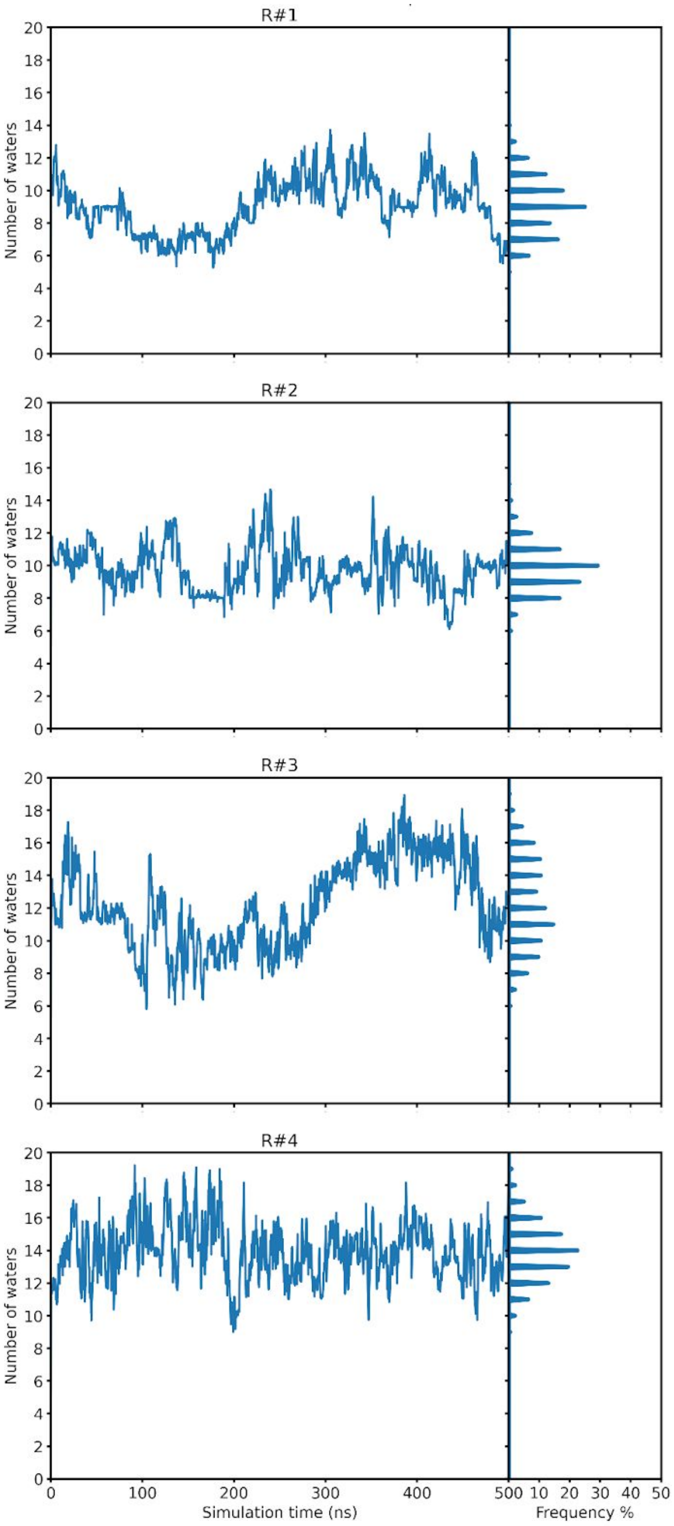

Figure S6. Number of water molecules in the binding site of the apo state of NPC1L1-NTD over simulation time.

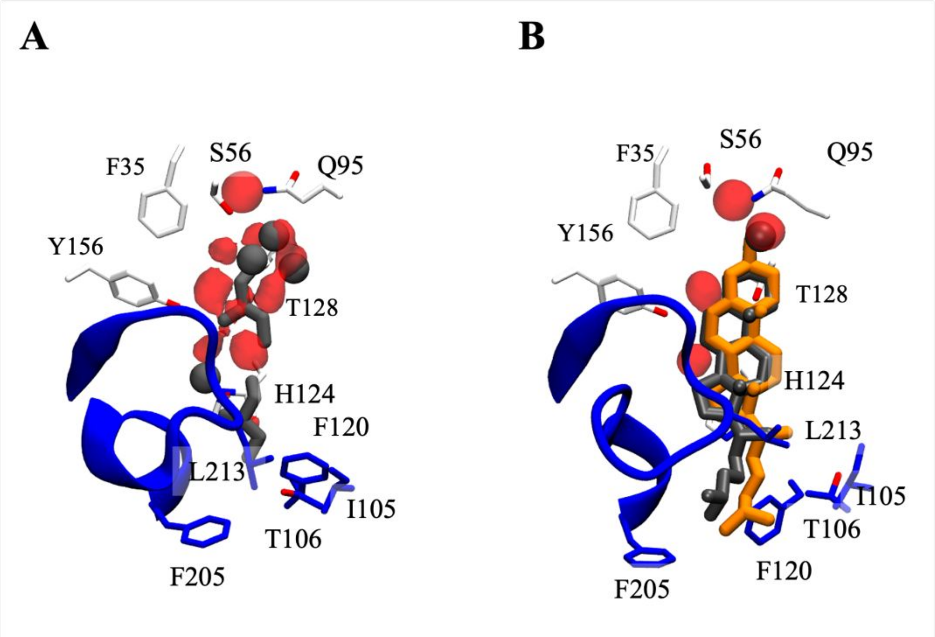

Figure S7. Overlap between the water network of NPC1L1-NTD with the X-ray structures of the NPC1-NTD. Close up views of the binding site in the (**A**) apo and (**B**) cholesterol-bound states. The occupancy of water molecules in the binding site over 50% of the simulation time is shown in the transparent red isosurface. The crystallographic molecules of water, cholesterol and glycerol found in the NPC1-NTD are shown as gray sticks and spheres. The NPC1L1-NTD is shown as white cartoon and sticks. The α8/β7 loop is shown as blue cartoon. The residues of the binding site of NPC1L1-NTD are shown as white and blue sticks.

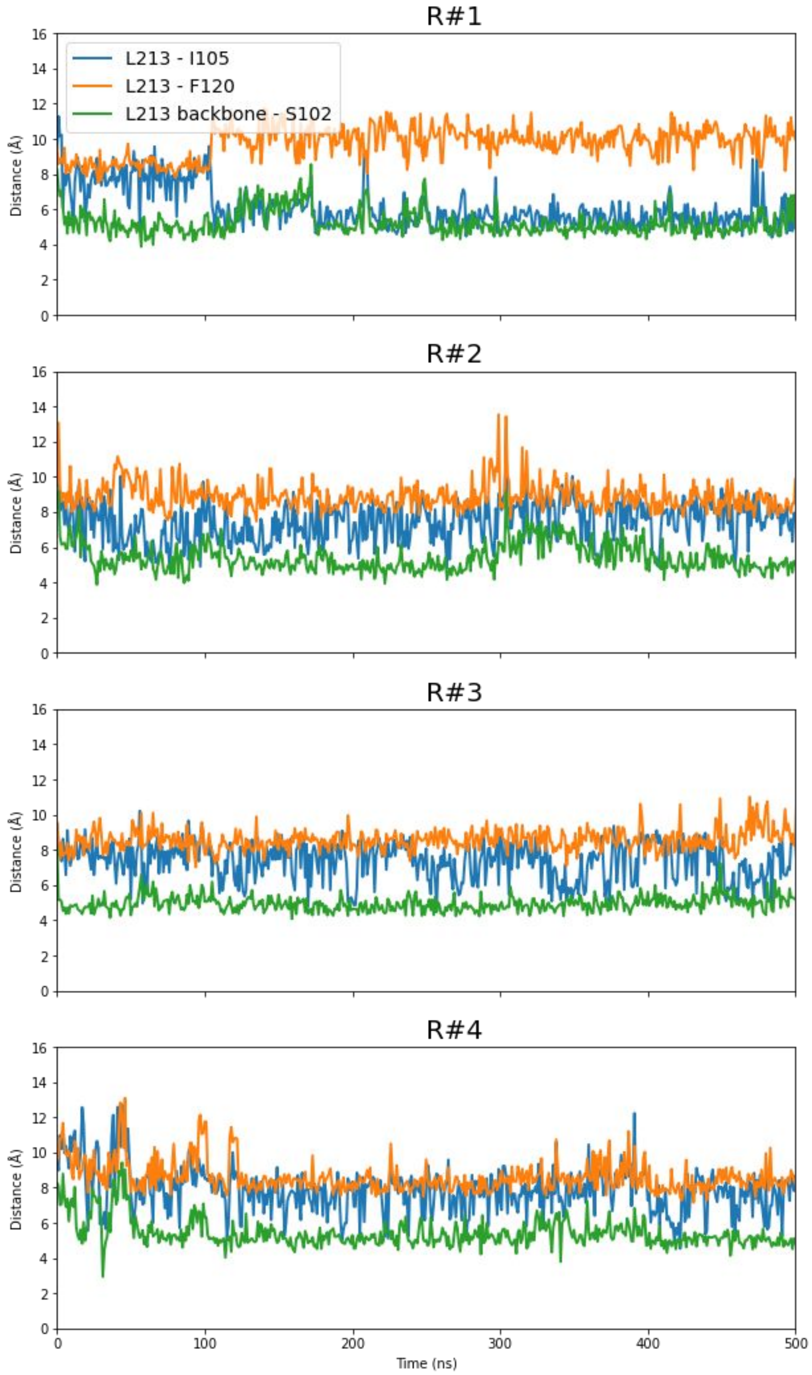

Figure S8. Selected interactions of L213 in the cholesterol-bound NPC1L1-NTD over simulation time. Blue and orange lines correspond to the distances of the CG atom of L213 to the CG atom of I105 and the center of the aromatic ring of F120 respectively. Green lines account for the H-bond interaction between the O atom of the backbone of L213 to the sidechain of S102.

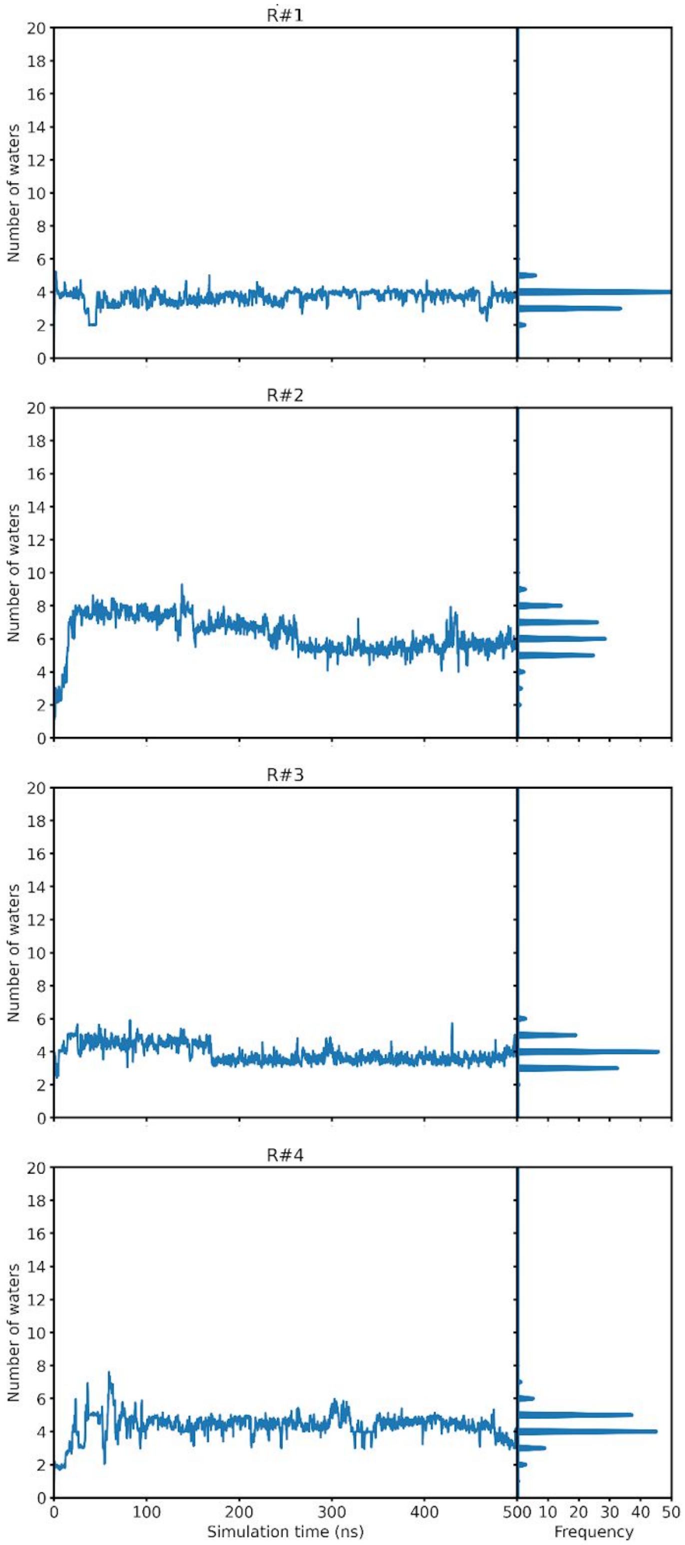

Figure S9. Number of water molecules in the binding site of the cholesterol-bound species of NPC1L1-NTD over simulation time.

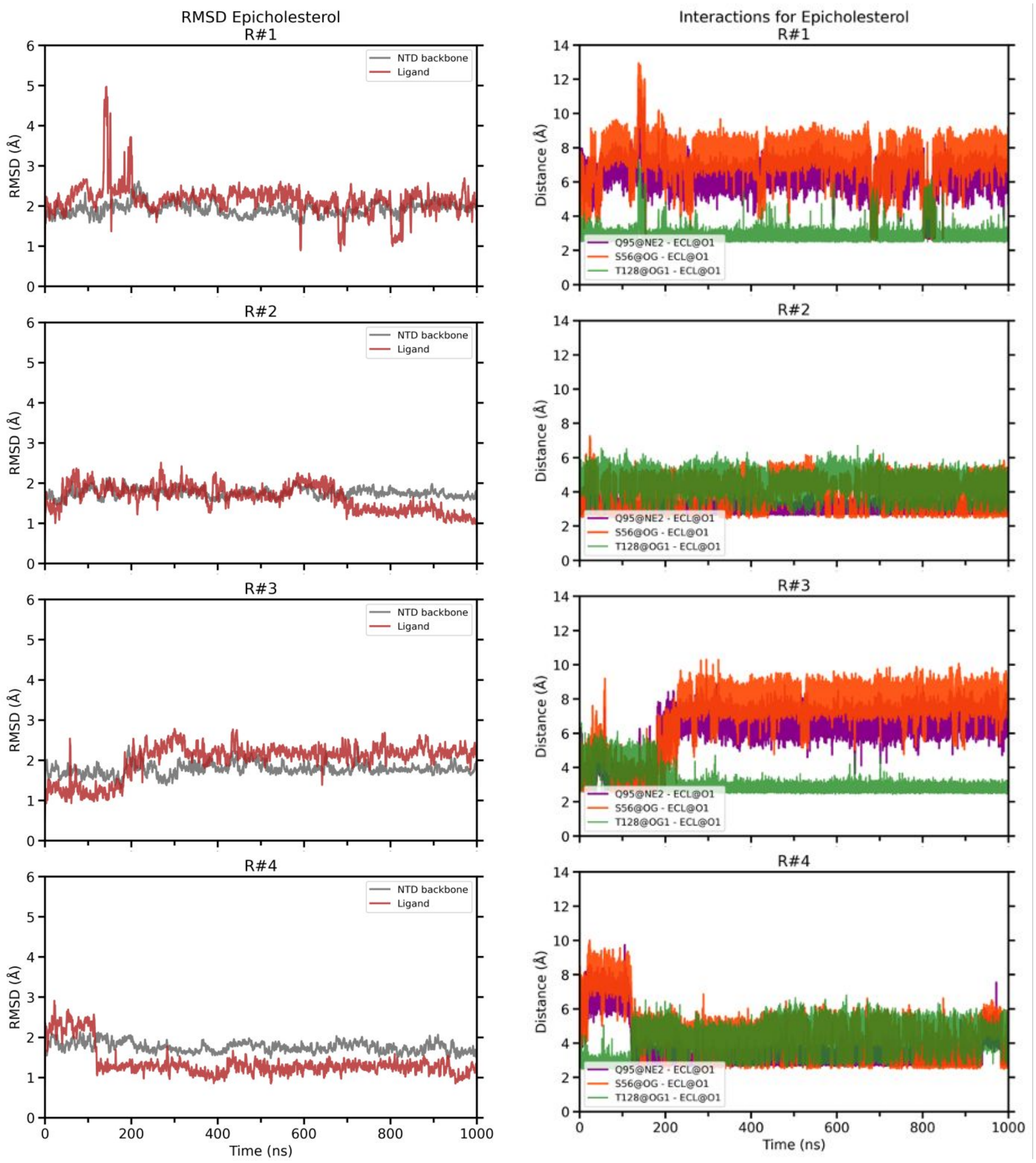

Figure S10. RMSD profiles of the epi-cholesterol-bound NPC1L1-NTD and H-bond interactions of the sterol over simulation time. (*Left*) Grey and red lines correspond to the RMSD of the backbone of the protein and heavy atoms of the ligand. (*Right*) Green, purple and orange lines correspond to the H-bonds between the hydroxyl moiety of the ligand and to the T128, Q95 and S35 sidechain.

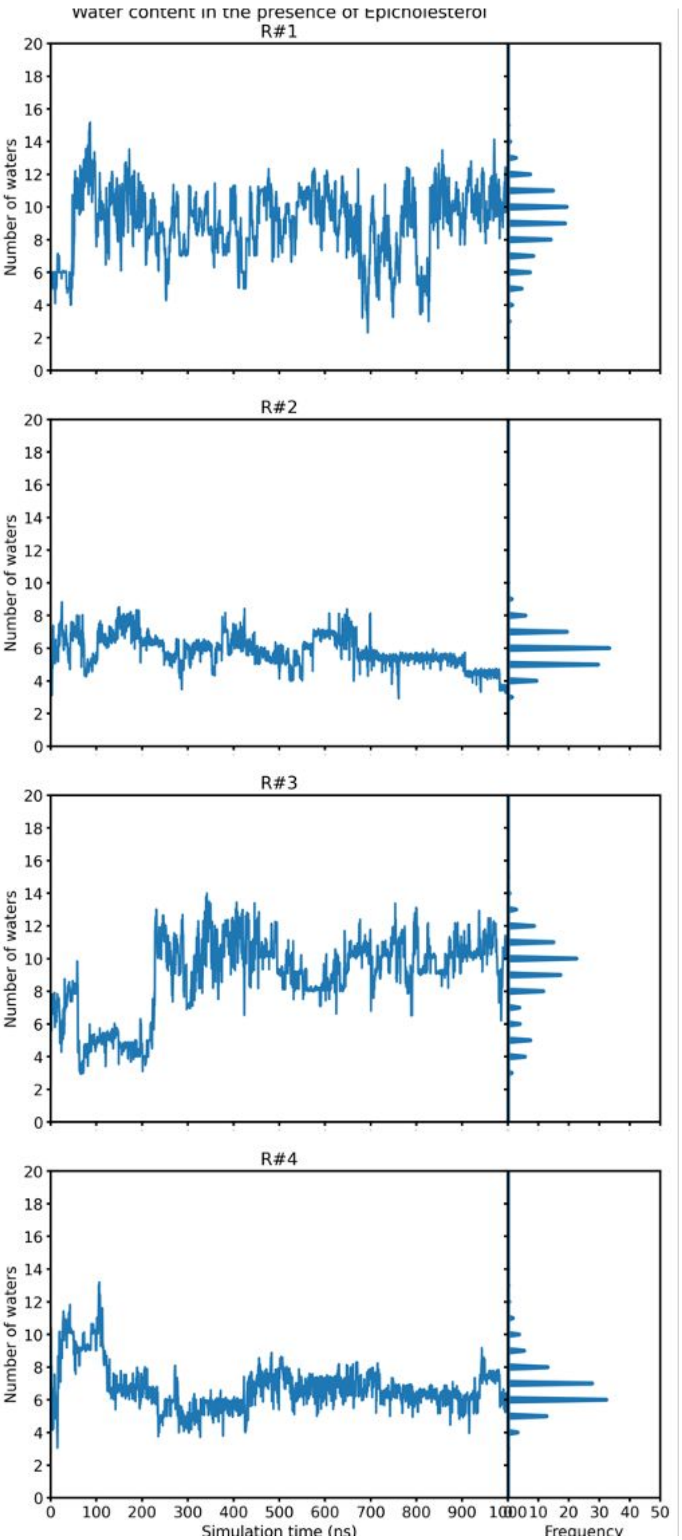

Figure S11. Number of water molecules in the binding site of the epi-cholesterol-bound species of NPC1L1-NTD over simulation time.

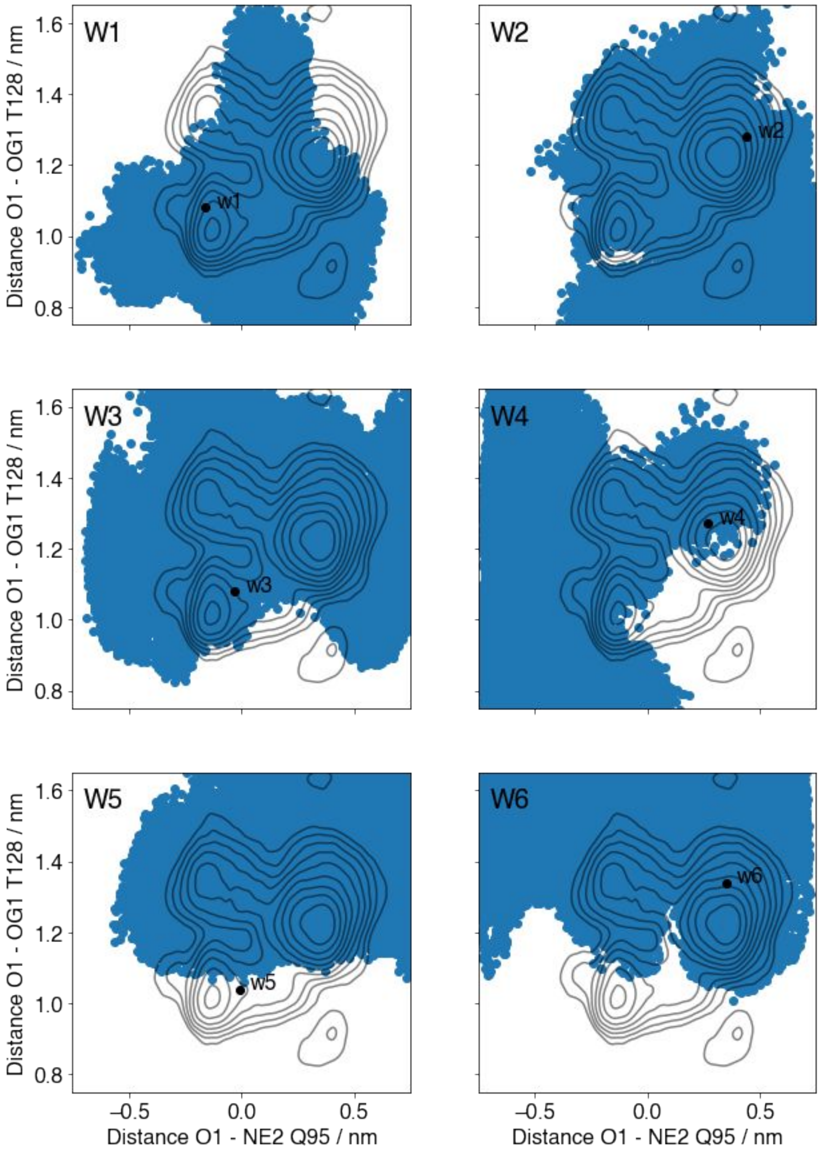

Figure S12. Representation of the two collective variables along the well-tempered metadynamics over the Collective Variable space. CV1 accounts for the difference of distances between the O atom of epi-cholesterol and the sidechains of residues Q95 and T128. CV2 accounts for the distance between the COM of the α8β7 loop and the α3 helix.

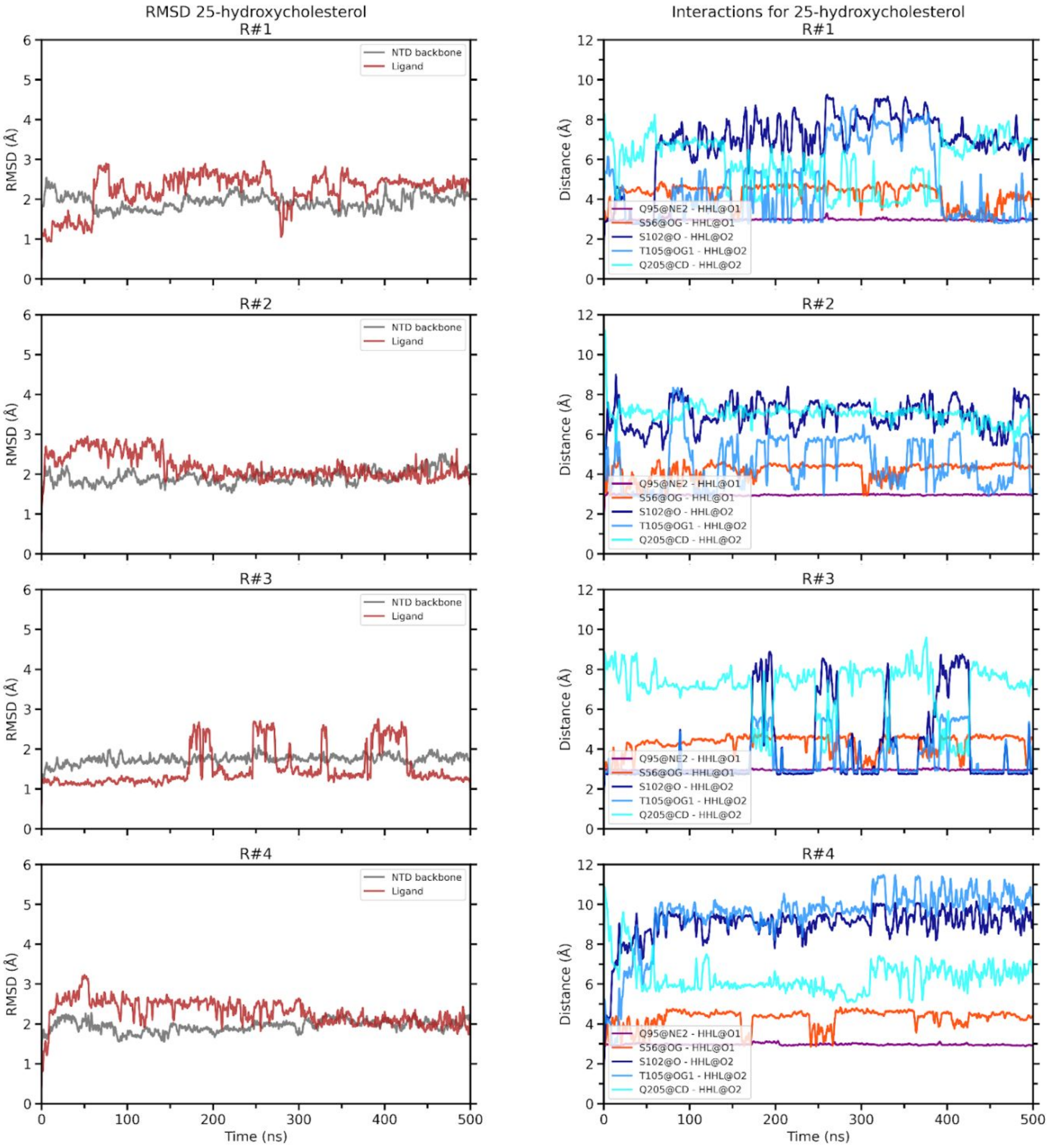

Figure S13. RMSD profiles of the 25-hydroxy-cholesterol-bound NPC1L1-NTD and H-bond interactions of the sterol over simulation time. (*Left*) Grey and red lines correspond to the RMSD of the backbone of the protein and heavy atoms of the ligand. (*Right*) Purple and orange lines correspond to the H-bonds between the hydroxyl moiety of the ligand and the sidechain of Q95 and S35. Blue lines show H-bond interaction of the 25-hydroxyl moiety.

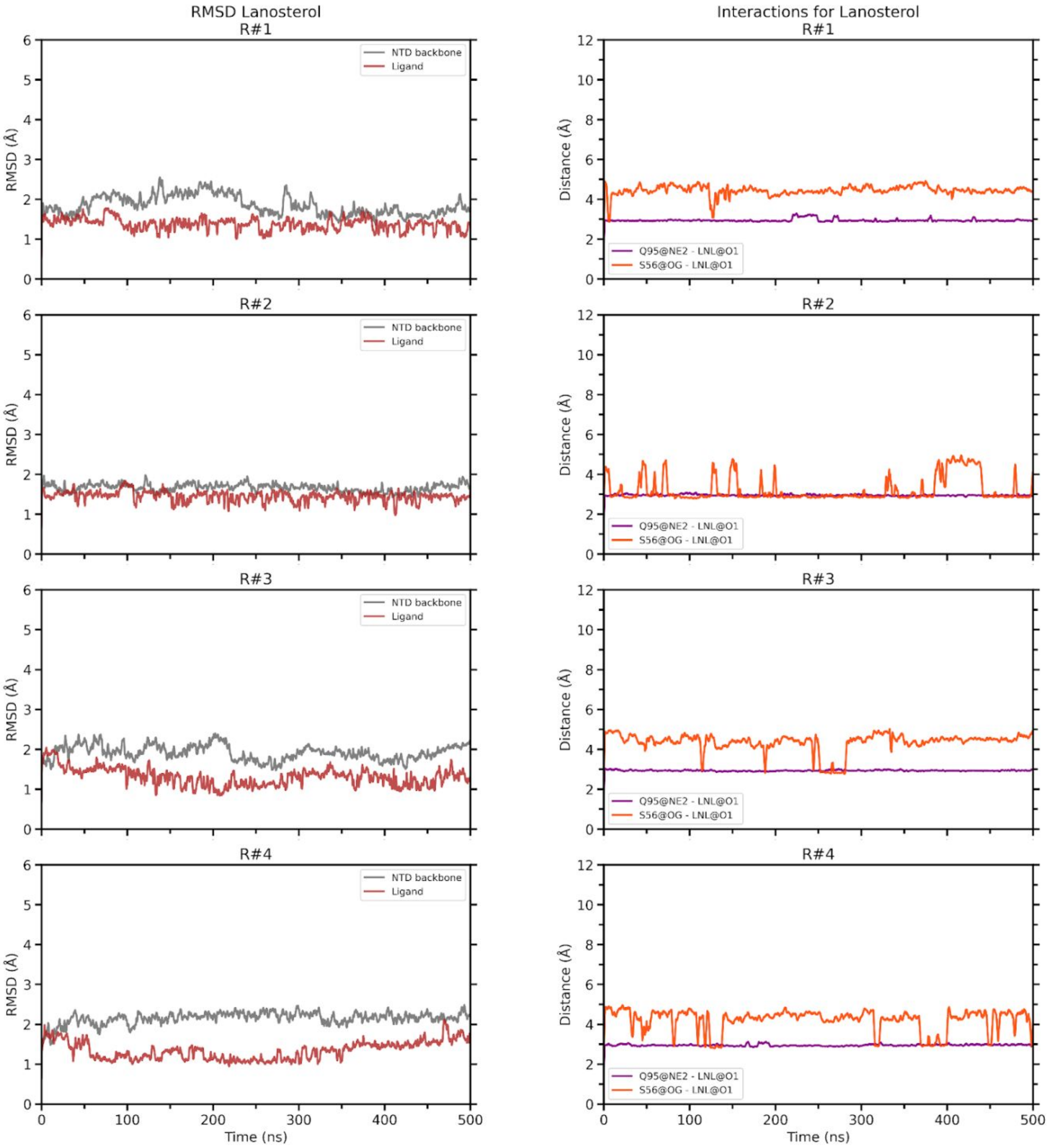

Figure S14. RMSD profiles of the lanosterol-bound NPC1L1-NTD and H-bond interactions of the sterol over simulation time. (*Left*) Grey and red lines correspond to the RMSD of the backbone of the protein and heavy atoms of the ligand. (*Right*) Purple and orange lines correspond to the H-bonds between the hydroxyl moiety of the ligand and the sidechain of Q95 and S35.

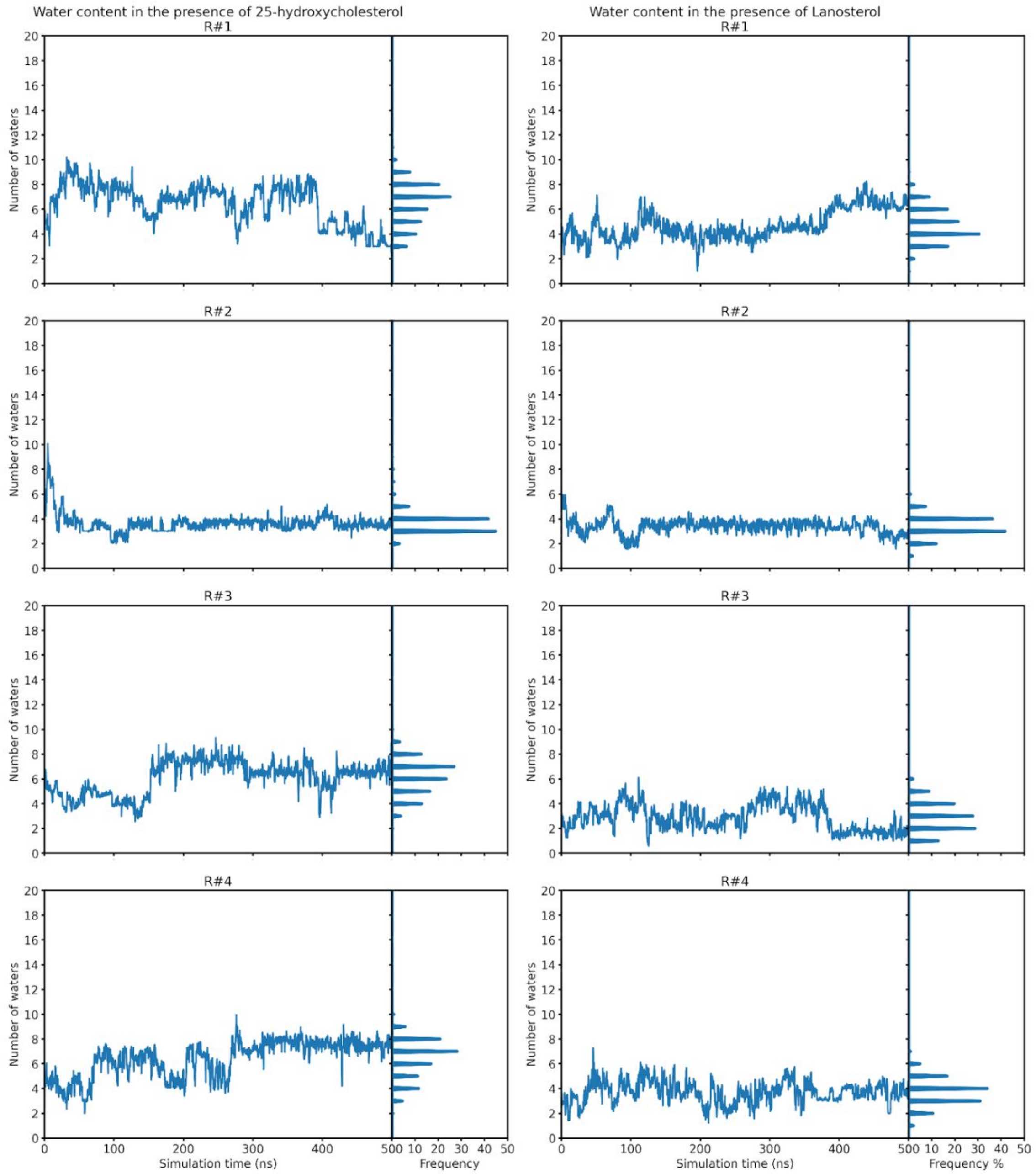

Figure S15. Number of water molecules in the binding site of (*left*) 25-hydroxy-cholesterol- and (*right*) lanosterol-bound species of NPC1L1-NTD over simulation time.

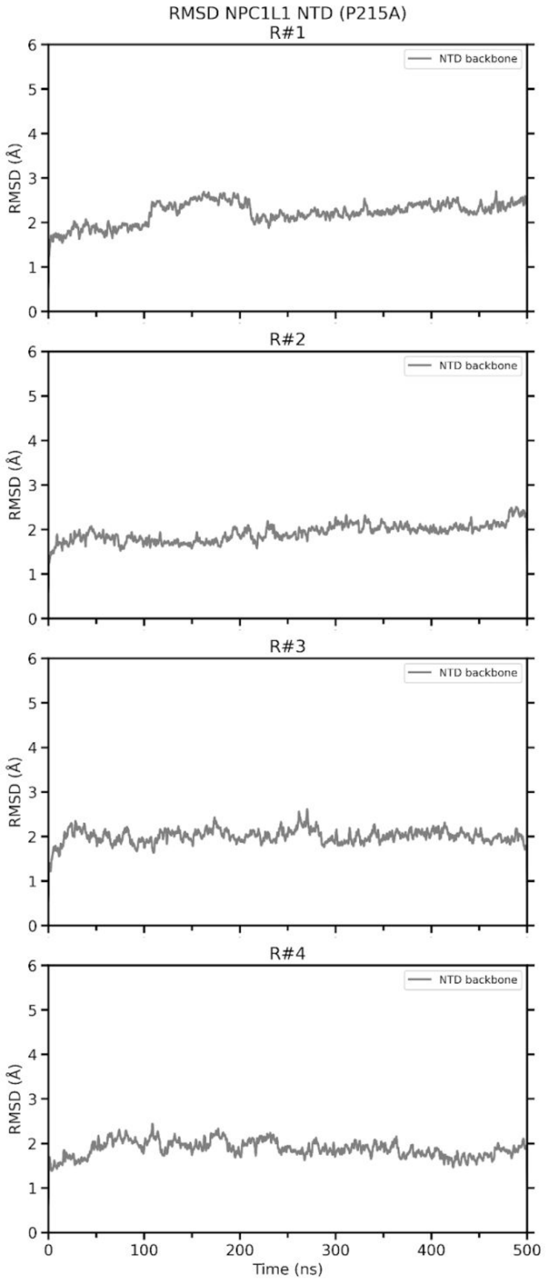

Figure S16. RMSD profiles of apo P215A NPC1L1-NTD over simulation time. Grey and red lines correspond to the RMSD of the backbone of the protein and heavy atoms of the ligand.

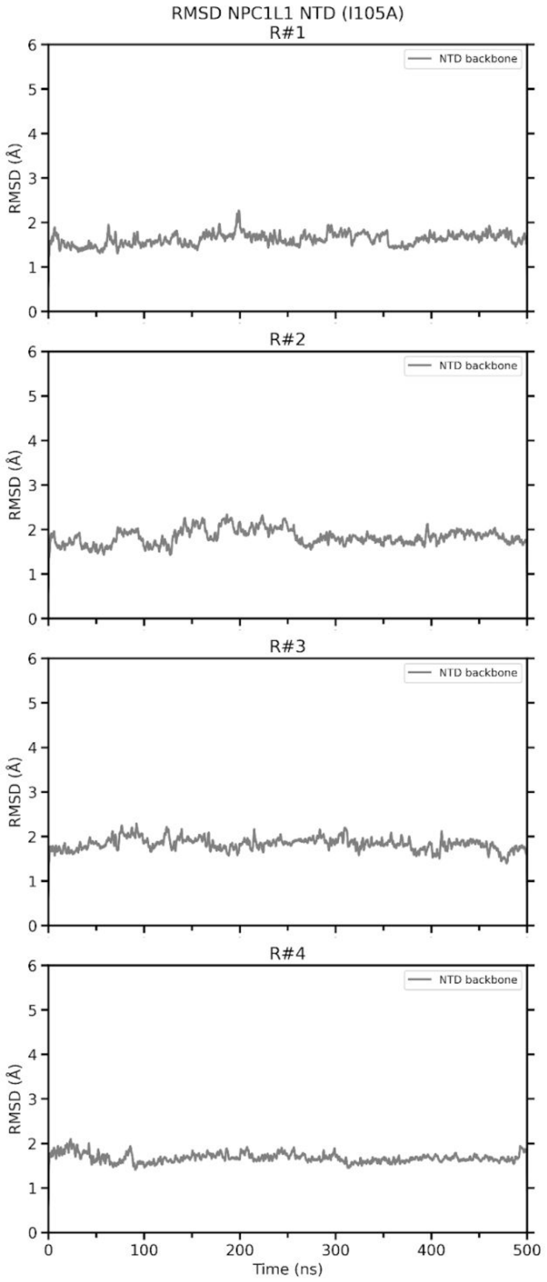

Figure S17. RMSD profiles of apo I105A NPC1L1-NTD over simulation time. Grey and red lines correspond to the RMSD of the backbone of the protein and heavy atoms of the ligand.

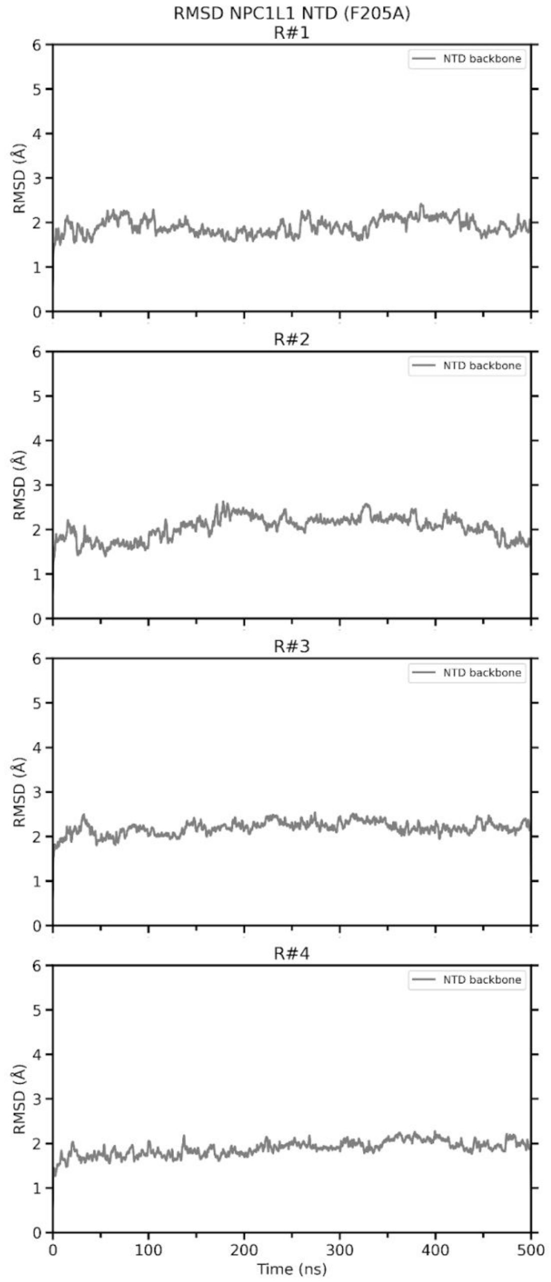

Figure S18. RMSD profiles of apo F205A NPC1L1-NTD over simulation time. Grey and red lines correspond to the RMSD of the backbone of the protein and heavy atoms of the ligand.

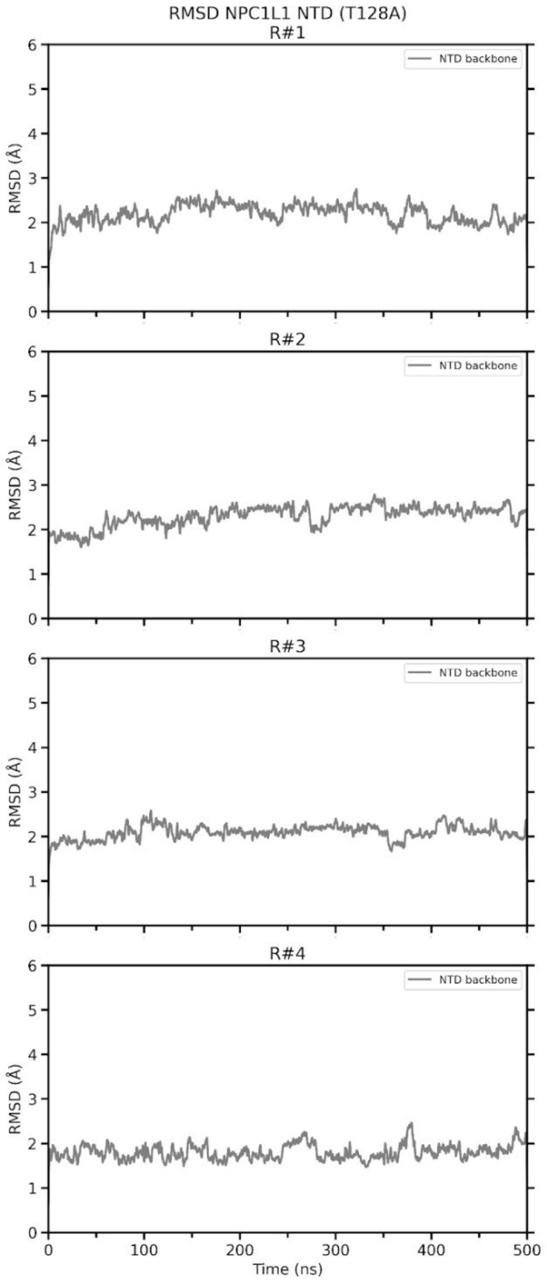

Figure S19. RMSD profiles of apo T128A NPC1L1-NTD over simulation time. Grey and red lines correspond to the RMSD of the backbone of the protein and heavy atoms of the ligand.

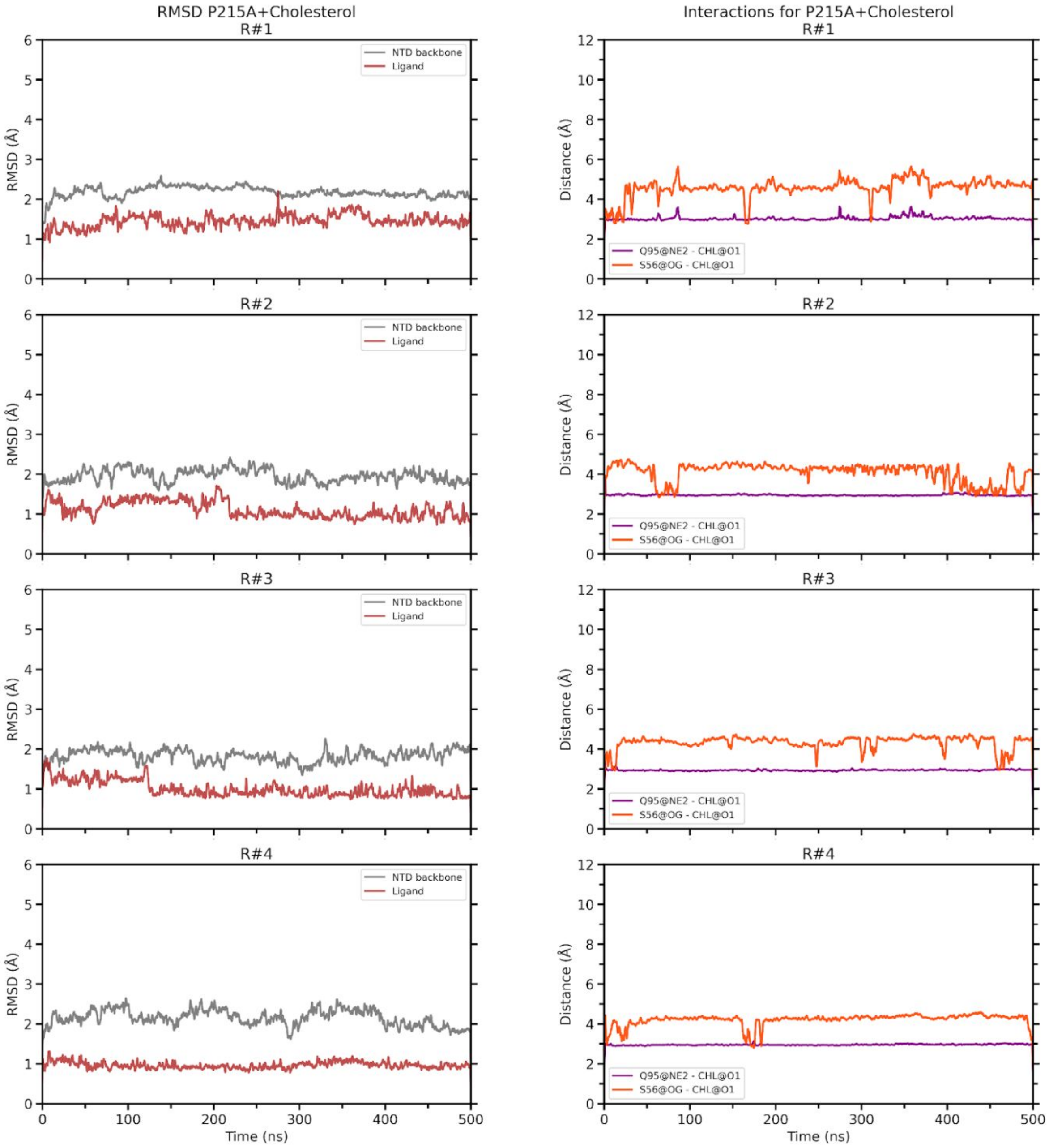

Figure S20. RMSD profiles of cholesterol-bound P215A NPC1L1-NTD and H-bond interactions of cholesterol over simulation time. Grey and red lines correspond to the RMSD of the backbone of the protein and heavy atoms of the ligand.

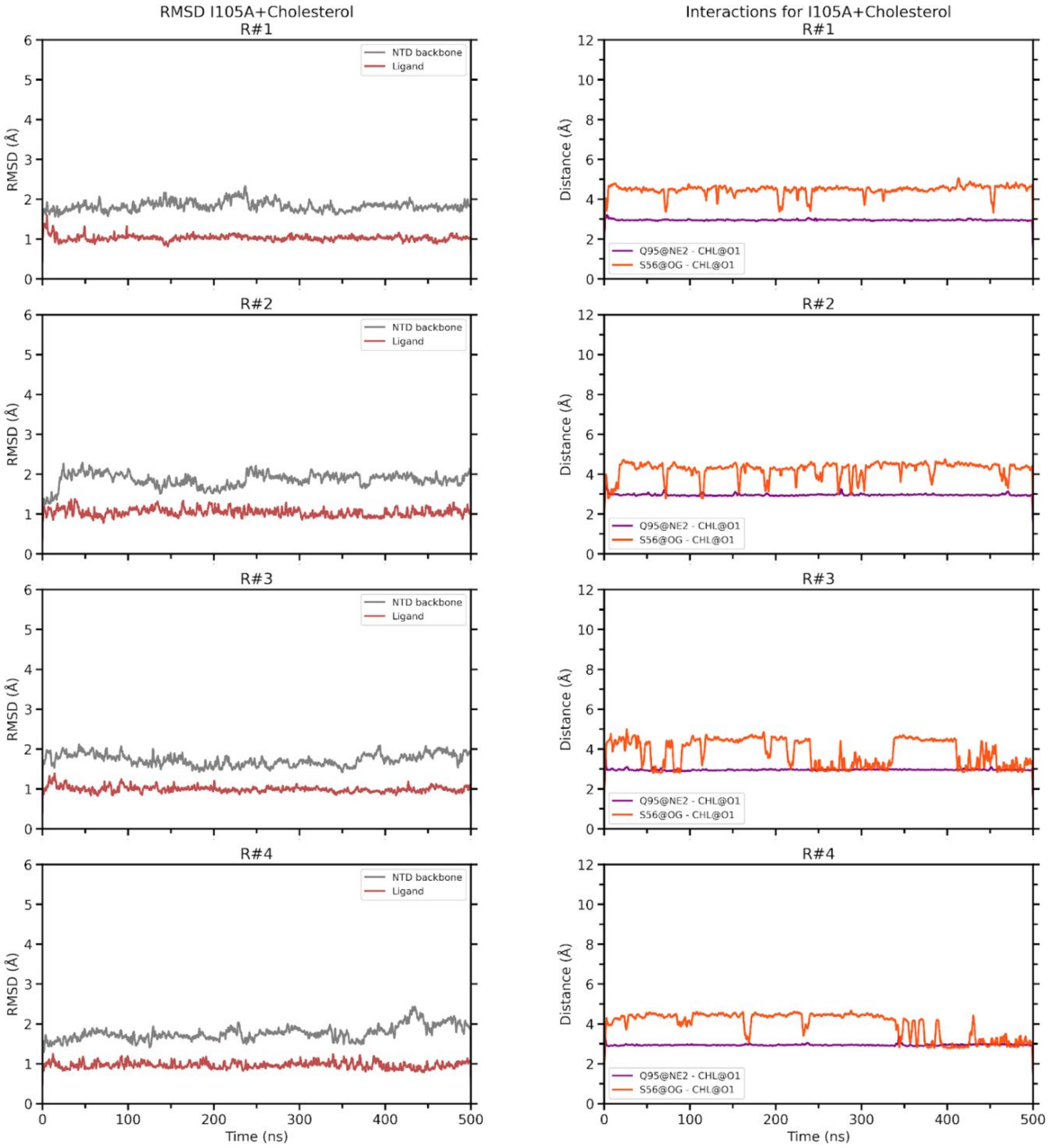

Figure S21. RMSD profiles of cholesterol-bound I105A NPC1L1-NTD and H-bond interactions of cholesterol over simulation time. Grey and red lines correspond to the RMSD of the backbone of the protein and heavy atoms of the ligand.

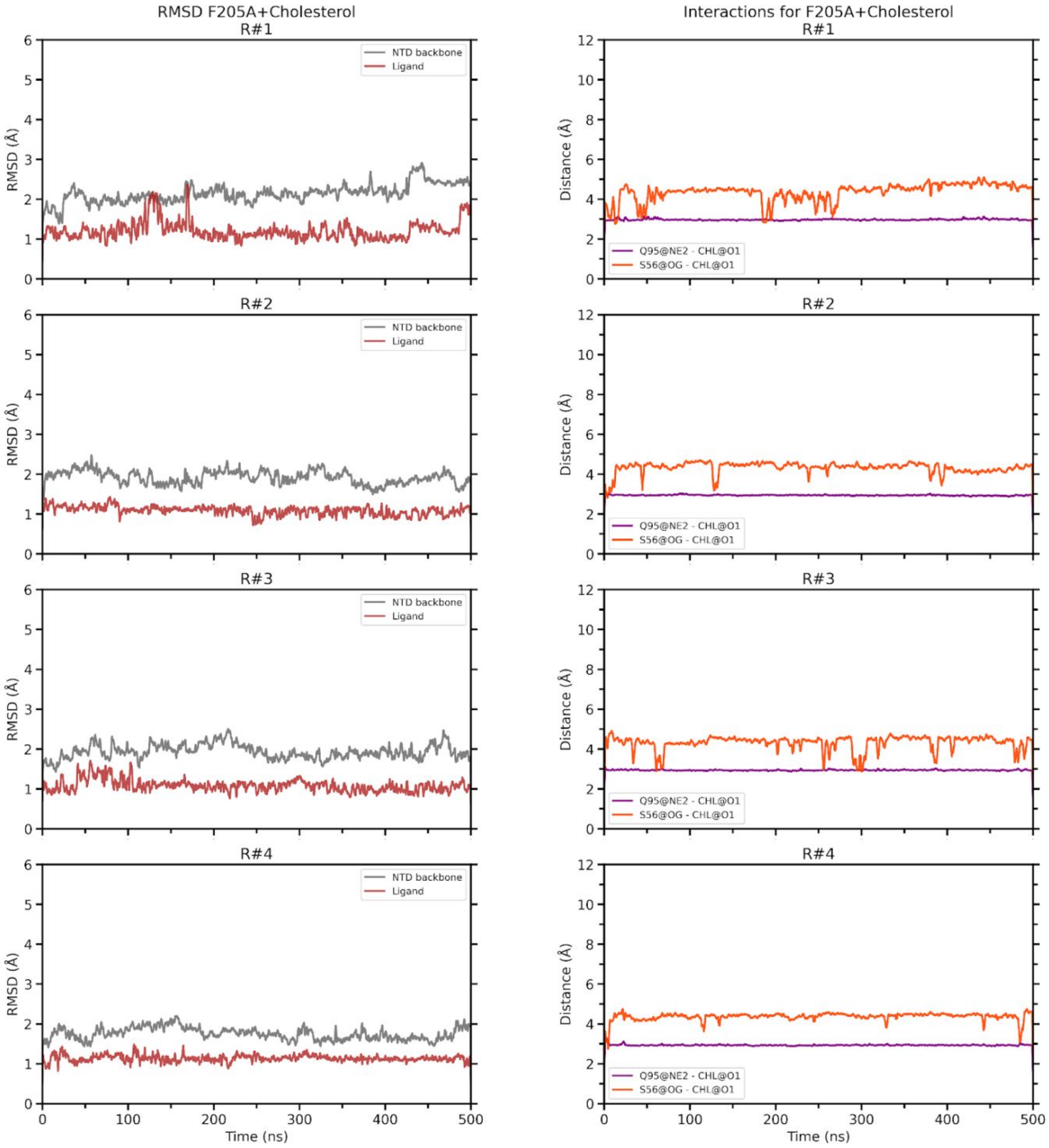

Figure S22. RMSD profiles of cholesterol-bound F205A NPC1L1-NTD and H-bond interactions of cholesterol over simulation time. Grey and red lines correspond to the RMSD of the backbone of the protein and heavy atoms of the ligand.

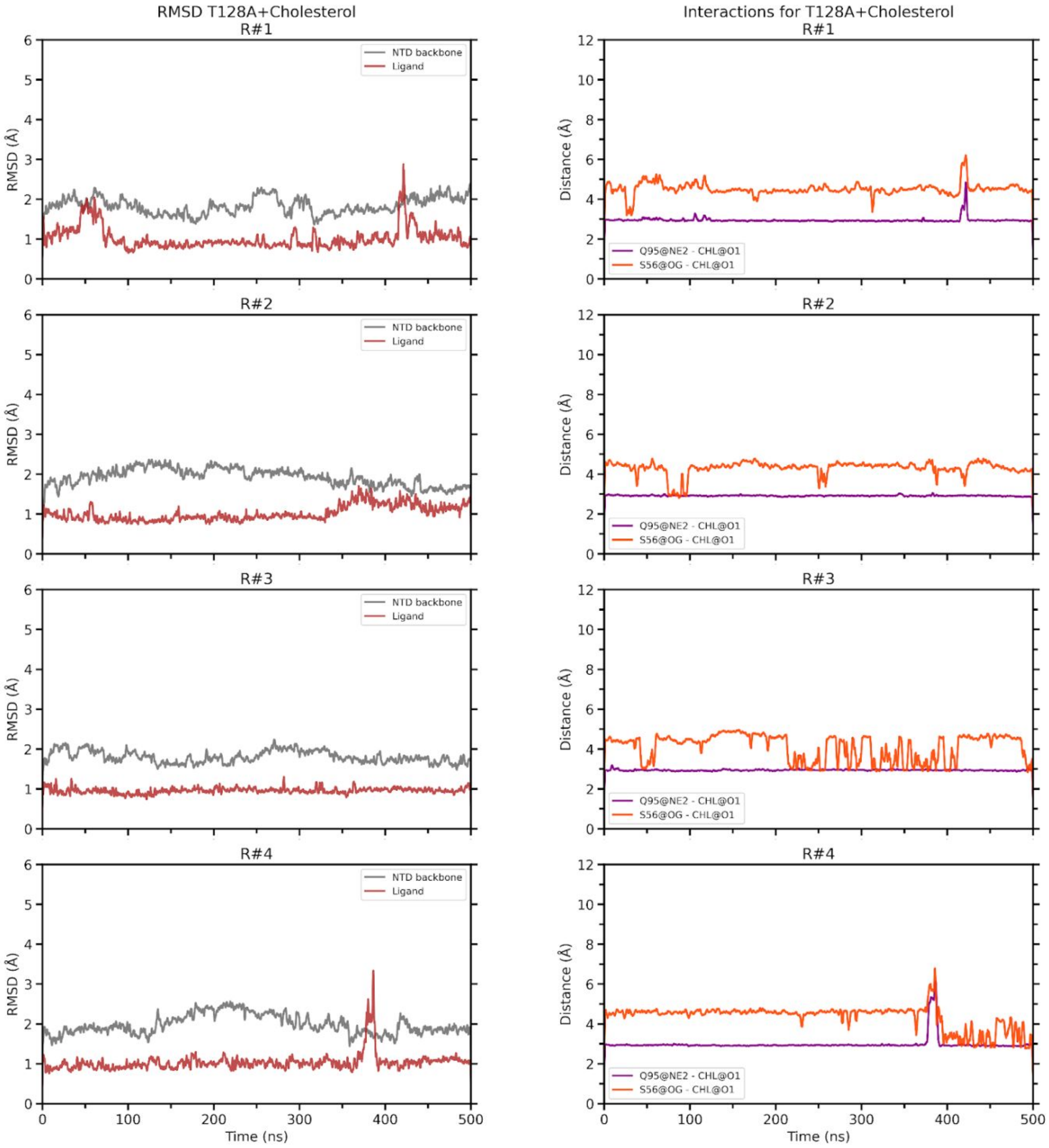

Figure S23. RMSD profiles of cholesterol-bound T128A NPC1L1-NTD and H-bond interactions of cholesterol over simulation time. Grey and red lines correspond to the RMSD of the backbone of the protein and heavy atoms of the ligand.

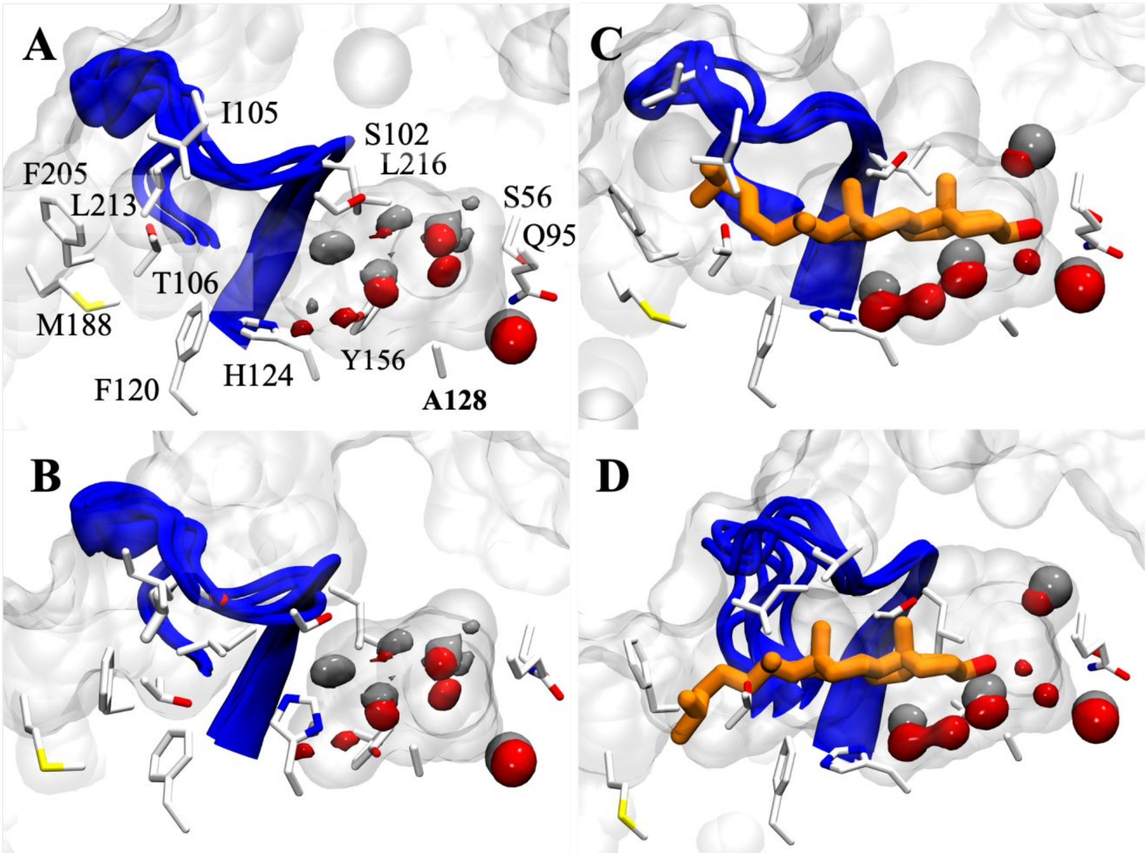

Figure S24. Superposition of the major conformations of the (**A,C**) apo and (**B,D**) cholesterol-bound states of the T128A mutant. (**A)** The major A2 and minor A1 (**C**) conformational states of the apo T128A mutant. **(B,D)** The two binding modes of cholesterol in the T128A mutants. The conformations sampled by the α8/β7 loop are shown as blue cartoon. The protein surface is shown as grey surface. The isosurface accounts for 50% of water occupancy in the binding site during the last 100 ns of each replicate (accumulated 400 ns of simulation time). The water occupancy for the wildtype protein and T128A mutant are shown as grey and red isocontours, respectively.

Table S1. Available structural information of the NPC1L1 protein on PDB database.

**NPC1L1** (Uniprot Q9UHC9 & Uniprot Q6T3U3)						
**PDB id**	**Method**	**Resolution Å**	**Spp**	**NTD-included**	**Ligand bound**	**Reference**
7DFW	EM	2.69	*Homo sapiens*	NO	Cholesterol	Long T. *et al* *Sci. Adv.* (**2021**) 7, eabh3997
7DFZ	EM	3.58	*Homo sapiens*	NO	Ezetimibe	
7DF8	EM	3.03	*Homo sapiens*	NO	---	
7N4V	EM	3.58	*Homo sapiens*	NO	Cholesterol	
7N4X	EM	3.33	*Homo sapiens*	NO	Cholesterol	
7N4U	EM	3.34	*Homo sapiens*	NO	α-tocopherol	
6V3F	EM	3.70	*Rattus norvegicus*	YES	Cholesterol	Huang CS. *et al* *Sci. Adv.* (**2020**) 6, eabb1989
6V3H	EM	3.50	*Rattus norvegicus*	YES	Ezetimibe analog	
3QNT	X-Ray	2.83	*Homo sapiens*	YES	---	Kwon HJ, *et al* *PLoS ONE* (**2011**) 6: e18722

Table S2. Available structural information of the NPC1 protein on PDB database.

**NPC1** (Uniprot O15118)						
**PDB id**	**Method**	**Resolution Å**	**Spp**	**NTD-included**	**Ligand bound**	**Reference**
8EUS	X-Ray	2.30	*Homo sapiens*	NO	---	Odongo L. *et al* *Acta Cryst. F* (**2023**) 79, 45–50.
6UOX	EM	4.02	*Homo sapiens*	YES	Itraconazole	Long T. *et al* *Nat Commun* (**2020**) **11**, 152.
6W5R	EM	3.60	*Homo sapiens*	YES	Cholesterol	Qian H. *et al* *Cell* (**2020**) *182*, 98-111
6W5S	EM	3.00	*Homo sapiens*	YES	Cholesterol	
6W5T	EM	3.70	*Homo sapiens*	YES	Cholesterol	
6W5U	EM	3.90	*Homo sapiens*	YES	Cholesterol	
6W5V	EM	4.00	*Homo sapiens*	YES	Cholesterol	
5U73	X-Ray	3.35	*Homo sapiens*	YES	---	Li, X. *et al* *Proc. Natl. Acad. Sci.* (**2017**), *114*, 9116-9121.
5U74	X-Ray	3.34	*Homo sapiens*	YES	---	
5KWY	X-Ray	2.41	*Homo sapiens*	NO	Cholesterol-sulfate	Li X. *et al* *Proc. Natl. Acad. Sci.* (**2016**) *113*, 10079–10084.
5JNX	EM	6.56	*Homo sapiens*	YES	---	Gong, X. *et al* *Cell* **(2016)** *165*, 1467–1478.
5HNS	X-Ray	2.45	*Homo sapiens*	NO	---	Zhao, Y. *et al* *FEBS Lett* (**2016**) 590: 605-612
5F18	X-Ray	2.00	*Homo sapiens*	NO	---	Wang, H. *et al* *Cell* (**2016**), *164*, 258–268.
5F1B	X-Ray	2.30	*Homo sapiens*	NO	---	
3GKH	X-Ray	1.81	*Homo sapiens*	YES	---	Kwon H. J. *et al* *Cell* (**2009**) *137*, 1213–1224.
3GKI	X-Ray	1.80	*Homo sapiens*	YES	Cholesterol	
3GKJ	X-Ray	1.60	*Homo sapiens*	YES	25-hydroxy-cholesterol	
3JD8	EM	4.43	*Homo sapiens*	YES	Cholesterol	To be published.

Table S3. Average distance (A) and preservation (% of simulation time) of the interactions between wild-type NPC1L1-NTD and the studied sterol molecules.

			Replicas			
**Complex**	**H-bond interaction**		**R1**	**R2**	**R3**	**R4**
**Cholesterol**	**Q95 and OH of sterol**	**Average distance**	2.9	2.9	2.9	2.9
		**%**	64	71	66	64
	**S56 and OH of sterol**	**Average distance**	2.8	2.8	2.8	2.8
		**%**	15	10	4	40
**25-hydroxycholesterol**	**Q95 and OH of sterol**	**Average distance**	2.9	2.9	2.9	2.9
		**%**	64	67	70	65
	**S56 and OH of sterol**	**Average distance**	2.8	2.8	2.8	2.8
		**%**	11	10	7	8
	**T106 and OH of aliphatic chain**	**Average distance**	2.8	2.8	2.8	2.8
		**%**	24	16	57	1
	**S102 and OH of aliphatic chain**	**Average distance**	2.8	2.8	2.7	2.8
		**%**	8	0	64	0
	**Q206 and OH of aliphatic chain**	**Average distance**	2.8	2.9	2.8	2.9
		**%**	23	0	9	0
**Lanosterol**	**Q95 and OH of sterol**	**Average distance**	2.9	2.9	2.9	2.9
		**%**	70	67	74	67
	**S56 and OH of sterol**	**Average distance**	2.8	2.8	2.8	2.8
		**%**	1	41	4	6

Table S4. Description of the main conformational states of the apo and cholesterol-bound systems found for the wildtype NPC1L1-NTD and its mutated variants.

	Conformation	Population %	Volume Å
**wildtype**	A1	24 ± 2	483 ± 116
	A2	75 ± 2	491 ± 145
	B1	97 ± 2	1025 ± 61
**I105A**	A2	76 ± 5	264 ± 43
	A4	18 ± 3	525 ± 44
	B1	93 ± 1	985 ± 52
**T128A**	A1	29 ± 1	459 ± 47
	A2	52 ± 4	434 ± 37
	A3	17 ± 3	347 ± 86
	B1	86 ± 1	935 ± 54
	B3	8 ± 1	968 ± 54
**F205A**	A1	12 ± 1	488 ± 123
	A2	35 ± 1	475 ± 126
	A3	31 ± 1	366 ± 43
	A4	23 ± 1	745 ± 95
	B1	90 ± 1	994 ± 45
	B3	4 ± 1	973 ± 72
**P215A**	A1	24 ± 1	361 ± 76
	A2	42 ± 1	390 ± 55
	A3	25 ± 2	444 ± 65
	A5	9 ± 1	288 ± 22
	B1	79 ± 1	902 ± 51
	B2	20 ± 1	941 ± 73
